# Agreement and longitudinal repeatability of established brain volumetry pipelines across healthy controls, multiple sclerosis, and Alzheimer's disease

**DOI:** 10.3389/fnins.2026.1917471

**Published:** 2026-10-16

**Authors:** Nedim Šišić, Muhamed Barakovic, Manon Edde, Antoine Théberge, Ann-Marie Beaudoin, Moussa Dumbia, Emmanuelle Lapointe, Maxime Descoteaux, Stefano Magon, Susanna Gobbi

**Affiliations:** 1 Roche Pharma Research and Early Development, Neuroscience and Rare Diseases Biomarkers, Basel, Switzerland; 2 Faculty of Mathematics, Natural Sciences and Information Technologies, University of Primorska, Koper, Slovenia; 3 Sherbrooke Connectivity Imaging Lab (SCIL), University of Sherbrooke, Sherbrooke, QC, Canada; 4 Division of Neurology, Department of Medicine, University of Sherbrooke (Université de Sherbrooke), Sherbrooke, QC, Canada; 5 Research Centre of the Sherbrooke University Hospital Centre (Centre de recherche du Centre hospitalier universitaire de Sherbrooke), Sherbrooke, QC, Canada; 6 Division of Neurology, Department of Medicine, Centre Hospitalier Universitaire de Sherbrooke, Sherbrooke, QC, Canada

**Keywords:** brain segmentation, volume measurements, MRI, MS, AD

## Abstract

**Introduction:**

Brain volume estimates are increasingly used as longitudinal endpoints in neuroimaging studies and clinical trials, but outputs from different segmentation pipelines may not be interchangeable. In this study, we performed a version-specific comparison of six established brain segmentation pipelines across healthy controls (HC; *n* = 20), people with multiple sclerosis (MS; *n* = 19), and people with Alzheimer's disease (AD; *n* = 50).

**Methods:**

FreeSurfer v6, FreeSurfer v7.2, SAMSEG, FastSurfer v1, SIENAX/SIENA-XL, and CAT12 were evaluated. The primary aims were to quantify systematic offsets and inter-pipeline agreement in normalized baseline volume estimates, assess within-pipeline repeatability and longitudinal change-from-baseline concordance, and determine whether longitudinal processing reduced variability relative to cross-sectional processing. Gray matter, white matter, hippocampus, and thalamus were selected and normalized to intracranial volume. Agreement was assessed using intraclass correlation coefficients, while longitudinal change was evaluated using mixed-effects models and repeatability metrics.

**Results:**

Baseline volume estimates differed significantly across pipelines in 11 of 12 cohort-by-tissue comparisons (FDR-adjusted *p* < 0.001), with the largest pipeline-dependent offsets observed for grey matter, hippocampus, and thalamus. Inter-pipeline agreement was generally strong for white matter and lower for gray matter and subcortical structures (hippocampus and thalamus), particularly in pathological cohorts. Change-from-baseline models showed no significant Pipeline × Timepoint interactions after FDR correction, indicating broadly parallel longitudinal trajectories across pipelines after baseline normalization. However, significant pipeline main effects persisted in selected tissues, especially in AD and subcortical regions, indicating residual differences in the magnitude of estimated change. Longitudinal processing reduced within-subject variability in selected settings, particularly for FreeSurfer-derived hippocampal and thalamic estimates, but did not uniformly improve stability across all pipelines or tissues.

**Discussion:**

Together, these findings show that systematic offset, absolute agreement, longitudinal change magnitude, and short-interval repeatability represent distinct properties of volumetric pipelines and vary by tissue, cohort, and processing mode. Longitudinal processing reduced variability but not uniformly, highlighting pipeline- and tissue-dependent differences relevant to the interpretation of longitudinal volumetric measurements.

## Introduction

Lonxgitudinal brain volume change is a critical endpoint in clinical trials, providing a quantitative assessment of disease progression and treatment effect in neurological disorders such as Alzheimer's disease (AD) and multiple sclerosis (MS). As therapies increasingly target disease modification, the ability to detect brain volume changes reliably is important.

Numerous segmentation algorithms have been developed to measure whole and regional brain volume loss. However, these tools vary substantially in terms of approach (ranging from voxel-based to surface-based and from cross-sectional to longitudinal), output, and computational demands. Moreover, results can be confounded by several factors such as scanner types, acquisition protocol, and operating system (Glatard et al., 2015). Thus, there is currently no consensus on which method offers the optimal balance of sensitivity and consistency in pathological cohorts (Goto et al., 2022).

Despite existing studies, direct head-to-head comparisons of multiple segmentation pipelines remain relatively limited, typically including few pipelines, small sample sizes, or only healthy cohorts (Glatard et al., 2015; Steenwijk et al., 2017; Guo et al., 2019; Bigler et al., 2020; Henschel et al., 2020; Bloch and Friedrich, 2021; Filip et al., 2022; Hedges et al., 2022; Haddad et al., 2023; van Nederpelt et al., 2023). Systematic differences in absolute volume, participant-level agreement, short-interval repeatability, longitudinal change estimates, and the benefit of dedicated longitudinal processing are often considered separately. Direct comparisons evaluating these properties within the same images and across both healthy and pathological cohorts remain relatively limited. Furthermore, the theoretical advantage of “longitudinal” processing modes compared with cross-sectional, which use subject-specific templates to reduce variability, remains under-evaluated in people undergoing significant pathological brain volume changes (Sederevičius et al., 2021; Cerri et al., 2023; Singh, 2023).

To address this gap, we conducted a version-specific head-to-head evaluation of six widely used brain segmentation pipelines across three populations: healthy control, people with MS and people with AD. As pipelines, FreeSurfer v6 and v7.2 (Fischl, 2012), SAMSEG (Puonti et al., 2016; Cerri et al., 2023), FastSurfer v1 (Henschel et al., 2020), SIENAX/-XL (Smith et al., 2002; Battaglini et al., 2018), and CAT12.8.2 (Gaser et al., 2024) were selected. No manual or phantom reference standard was available, thus the study was designed to assess volumetric agreement, systematic offsets, and longitudinal repeatability/concordance rather than absolute segmentation accuracy. Primary aims were to (1) quantify inter-pipeline agreement and systematic offsets in cross-sectional normalized volume estimates; (2) quantify within-pipeline repeatability and inter-pipeline concordance of change-from-baseline estimates; and (3) assess whether longitudinal processing modes reduce variability or improve concordance relative to cross-sectional processing. Secondary aims were to explore whether these patterns differed by tissue type and cohort, and to summarize practical considerations for consistent pipeline use in longitudinal neuroimaging studies.

## Methods

### Participants

We performed the segmentation pipelines' comparison on three datasets. A dataset of healthy controls (Edde et al., 2023b) and participants with multiple sclerosis (Edde et al., 2023a) recruited at University of Sherbrooke and the Centre Hospitalier Universitaire of Sherbrooke (CHUS) and a subset of the placebo arm of the AD phase III clinical trial Graduate I (Bateman et al., 2023; Bittner et al., 2025). Demographic information is reported in Table 1.

**Table 1:** Demographic info.

Cohort	Sherbrooke HC	Sherbrooke MS	Graduate
Sample size	19	20	50
Age	36 ± 4.7 years	37.11 ± 6.8 years	73 ± 7 years
Sex	4 male	4 male	24 male
	16 female	15 female	26 female
Pathology status	Healthy	Relapsing-remitting multiple sclerosis	50% MCI due to AD
			50% mild AD dementia

MCI, mild cognitive impairment.

#### Sherbrooke datasets

The Sherbrooke cohort consists of two datasets. The healthy controls dataset contains MRI of 19 healthy adults (mean age: 36 ± 4.7 years; 4 male/16 female). Eligibility criteria included no history of brain disease or injury. MRI data were acquired using Ingenia (Philips Healthcare) 3T scanner with 32-channel head coil. Participants underwent five MRI sessions (acquisition time of 4 min 20 s) over a 6-month period at 4-week intervals (+/- 1 week). The 3D T1-weighted (T1w) MPRAGE were acquired axially at 1.0 mm isotropic resolution, repetition time (TR), echo time (TE) and inversion time (TI) 7.9, 3.5 and 950 ms, respectively. The field-of-view (FOV) was 224 × 224 mm^2^, yielding 150 slices with a flip angle of 8°.

The second dataset contains MRI from 20 participants (mean age: 37.11 ± 6.8 years; 4 male/15 female) with relapsing-remitting multiple sclerosis (RR-MS), age and gender matched with the healthy cohort. The MRI dataset of five scans performed at 4-week intervals (±1 week) was acquired on the same scanner using a similar protocol to that of the healthy cohort. Additionally, a fluid-attenuated inversion recovery (FLAIR) image was acquired for each RR-MS participant for white matter lesion segmentation (see Table 2). Gradient distortion correction was applied to all scans in both datasets.

**Table 2:** Scan geometry and scanner parameters for the MRI images acquisition.

Cohort	Sherbrooke		Graduate		
Image type	T1	T2 FLAIR	T1		
Scanner type	Ingenia		Philips	Siemens	GE
	(Philips Healthcare) 3T		1.5T/3T	1.5T/3T	1.5T/3T
Duration	4 min 20 s	4 min 20 s	5–10 min	5–10 min	5–10 min
FOV (mm)	224 × 224	250 × 250	240 × 240	240/270	240/270
Number of slices	150	326	160–176	160–176	160–176
Voxel size (mm)	1 × 1 × 1	0.74 × 0.74 × 0.56 (reconstructed)	1.2 × 1.2 × 1.2	1.2 × 1.2 × 1.2	1.2 × 1.2 × 1.2
Direction	Axial	Sagittal			
Phase-encoding			A/P	A/P	
Sequence type	FFE–TFE	IR–TSE	3D TFE	Turbo FLASH	Fast SPGR
TR (ms)	7.9	4,800	Shortest	2,400/2,300	9/6
TE (ms)	3.5	340	Shortest	3.5/2.98	Min full
TI (ms)		1,650			
Flip angle (°)	8	90	8/9	8/9	8/11

#### Graduate I dataset

Participants were selected from the placebo arm of the randomized, double-blind, pivotal Phase III GRADUATE I trial (NCT03444870) to create a test dataset with complete longitudinal MRI data. From the 485 participants who received placebo, we identified participants with scans available at all four time points and manually selected 25 participants with mild cognitive impairment due to AD and 25 with mild dementia due to AD. The resulting cohort comprised 50 participants (mean age: 73 ± 7 years; 24 male/26 female). Selection was based only on the diagnostic group and availability of complete longitudinal MRI data; no imaging-derived measurements or study outcomes were considered. 3D T1w images were acquired by different scanners (GE, Philips, or Siemens) with field strength of 1.5T or 3T (depending on the site), a spatial resolution of 1.2 mm isotropic, and acquisition time between 5 and 10 min (see Table 2). Gradient distortion correction was applied to all scans. A centralized imaging vendor implemented a uniform acquisition protocol, with parameters customized according to scanner manufacturer, model, hardware/software configuration, and field strength. Harmonization procedures included site and technologist training, pre-study phantom qualification, review of the first participant scan at each site, and scan-level quality control of participant positioning, anatomical coverage, contrast, signal-to-noise ratio, spatial resolution, artifacts, and protocol adherence. Follow-up scans were compared with the baseline scan for consistency in scanner type, acquisition parameters, contrast, dynamic range, and resolution. All four MRI sessions for each participant were acquired on the same scanner, using an unchanged protocol and consistent positioning, at baseline and weeks 48, 104, and 116. Although residual differences among scanners and field strengths cannot be completely excluded and may have affected cross-sectional volume estimates, their influence on within-participant longitudinal estimates was minimized by these procedures.

### Image analysis

For the Sherbrooke cohorts, T1-weighted (T1w) images underwent quality control using the dMRIqc workflow (https://github.com/scilus/containers-dmriqc). Images were screened for uncorrectable artifacts, incorrect field of view, excessive noise, corrupted images, and incomplete brain coverage. Full details of the procedure are provided in the Sherbrooke dataset documentation (https://high-frequency-mri-database-supplementary.readthedocs.io/en/latest/pipeline/mri_processing.html#quality-control).

For the GRADUATE cohort, T1w images underwent a two-stage quality-control procedure before inclusion in the present analysis. First, incoming MRI examinations were assessed for protocol adherence and image quality by trained imaging technologists, with radiologist review available for equivocal cases. Assessment included verification of acquisition parameters and visual evaluation of participant positioning, anatomical coverage, signal-to-noise ratio, gray/white-matter contrast, intensity uniformity, spatial resolution, geometric accuracy, and imaging artifacts. For longitudinal examinations, follow-up scans were additionally compared with the corresponding baseline examination for consistency in scanner type, acquisition parameters, image contrast, dynamic range, resolution, and anatomical coverage. A second QC step was performed using MRIQC v23.1 (https://mriqc.readthedocs.io/en/latest/about.html) followed by visual review. MRIQC-based screening considered signal-to-noise ratio, contrast-to-noise ratio, entropy-focus criterion, and coefficient of joint variation. Potentially atypical images were identified using Tukey's 1.5 × IQR criterion and subsequently reviewed visually; this criterion was used as a screening threshold rather than as an automatic exclusion rule. The 50 participants included in the present study were selected from images that had passed these QC procedures.

Images were segmented using FreeSurfer v6.0, FreeSurfer v7.2, SAMSEG, FastSurfer v1, SIENAX, SIENA-XL, and CAT12.8.2. Both cross-sectional and longitudinal models of all pipelines were implemented except for FastSurfer, for which the longitudinal model was not available at the time of the analysis. In the MS cohort, lesion filling was performed before the segmentation. Gray matter (GM) and white matter (WM) volumes were estimated for all pipelines, although the anatomical and tissue-class definitions differed between methods, as detailed below. Hippocampal and thalamic volumes were not available from SIENAX/-XL and were therefore evaluated only for FreeSurfer, SAMSEG, FastSurfer, and CAT12. For the FreeSurfer-like pipelines, hippocampal and thalamic structures contributed to the global GM measure, whereas CAT12 regional volumes were derived separately from the Neuromorphometrics atlas and were not subsets of the CAT12 global GM estimate. Analyses of subcortical structures were restricted to the hippocampus and thalamus because of their established use as volumetric biomarkers in neurodegenerative disease. Volume estimates were expressed as a percentage of intracranial volume (ICV) to provide a common normalization across pipelines.

All completed segmentation outputs were visually inspected for gross errors, including brain-extraction failures, missing tissue, major pial or white-matter surface errors, and subcortical mislabeling. The assessment was performed by two members of the study team, with cases showing outlying volumetric measurements receiving additional review by two further members. The assessment was not blinded, and formal inter-rater agreement was not evaluated. No manual corrections of completed segmentations were performed. Processing failures and QC findings were recorded by cohort, pipeline, and processing mode.

The availability of tissue measures and processing modes across pipelines is summarized in Table 3.

**Table 3:** Availability of volumetric measures and processing modes across segmentation pipelines.

Pipeline	GM	WM	Hippocampus	Thalamus	XSEC	LONG
FreeSurfer v6	✓	✓	✓	✓	✓	✓
FreeSurfer v7.2	✓	✓	✓	✓	✓	✓
SAMSEG	✓	✓	✓	✓	✓	✓
FastSurfer v1	✓	✓	✓	✓	✓	–
SIENAX/SIENA-XL	✓	✓	–	–	✓	✓
CAT12.8.2	✓	✓	✓	✓	✓	✓

This table summarizes the tissue and regional volume measures included in the analysis and the availability of repeated cross-sectional (XSEC) and longitudinal (LONG) processing for each pipeline.

#### Lesion filling

To avoid potential variations due to lesions and improve the accuracy of brain tissue segmentation (Magon et al., 2014), lesion filling was performed on the MS dataset. For this dataset, 3D FLAIR images were acquired at each MRI visit for lesion segmentation. Full details of the lesion-segmentation protocol can be found in the Supplementary Material of Edde et al. (2023a,b). Briefly, FLAIR images were co-registered to T1w space using ANTs, and lesions were manually segmented in ITK-SNAP v3.6 by two raters (ME and EL). Lesions were identified mainly on FLAIR, with lesion borders delimited on T1w images, switching between contrasts when needed. Segmentations were first performed in the axial plane and checked in sagittal and coronal planes, including adjacent slices to assess 3D lesion shape. For follow-up scans, FLAIR and T1w images were registered to baseline and the previous lesion segmentation was used as reference, adding new or enlarged lesions (diameter ≥ 2 mm).

Lesion filling was then performed using FSL 6.0.4-based tools. Lesion masks registered to T1w space were binarized. For each T1w image, a brain mask was generated using FreeSurfer recon-all -autorecon1, converted to NIfTI, binarized, hole-filled, and transformed back to T1w space. The T1w image was skull-stripped using this brain mask and segmented using FSL FAST. The FAST white matter partial-volume map was thresholded to generate a binary WM mask, which was used to constrain lesion filling to WM-like intensities. Lesion-mask components not overlapping this WM mask were removed before lesion filling. Lesion filling was performed using the original T1w image, the cleaned lesion mask, and the FAST-derived WM mask, so that lesion voxels were filled with intensities similar to neighboring WM tissue (Zhang et al., 2001). This method has been shown to improve segmentation and volume estimates in participants with MS (Battaglini et al., 2012). Finally, the intensity outliers below 5% and above 95% were removed from the filled lesions to reduce contrast and the resulting images were visually checked by NS.

#### FreeSurfer

Cross-sectional and longitudinal FreeSurfer versions 6.0 and 7.2 were used to segment the brain into GM, WM, and subcortical structures (Fischl et al., 2002, 2004). For scans acquired at 3T, recon-all was called with the −3T flag. Correction for enlarged ventricles was applied in the AD cohort. After cross-sectional processing of each timepoint, an unbiased within-participant template was created using all available timepoints. Each timepoint was then reprocessed using FreeSurfer's longitudinal stream, which incorporates information from both the within-participant template and the corresponding cross-sectional reconstruction (Reuter et al., 2012). Thus, the longitudinal outputs are derived in part from the preceding cross-sectional processing and are not independent of the cross-sectional results. GM volume was defined using TotalGrayVol, which includes cortical, subcortical, and cerebellar GM but excludes the brainstem. WM was defined using a study-specific composite comprising CerebralWhiteMatterVol, left and right cerebellar WM, and the FreeSurfer Brain-Stem volume. This broader composite was used to provide anatomical coverage more comparable to pipelines that estimate global GM and WM from tissue-class segmentation. FreeSurfer v6 was retained as a clinically relevant legacy reference because many existing longitudinal datasets and version-specific biomarker definitions were developed using this release, and transition to a newer version may require complete reprocessing or formal bridging to preserve compatibility, as illustrated for FreeSurfer-derived striatal thresholds in Huntington's disease staging (Knights et al., 2024). FreeSurfer v8 was not included because the present study focused on the transition from the established FreeSurfer v6 to FreeSurfer v7.2. This comparison is clinically relevant because FreeSurfer-derived volumetric outputs can differ across software versions, and previous work has shown that version compatibility should not be assumed (Hedges et al., 2022; Haddad et al., 2023). Evaluation of FreeSurfer v8 was therefore considered an important next step but outside the scope of the current analysis.

#### SAMSEG

From FreeSurfer 7.2, the cross-sectional and longitudinal SAMSEG integration was used (Puonti et al., 2016; Cerri et al., 2023). SAMSEG performs voxel-based brain segmentation into GM, WM, and subcortical structures. The lesion filling option was disabled. For the longitudinal stream, the “samseg-long” command was executed to create the within-participant template and then the “recon-all” surface pipeline was run on this template. Specifically, all available timepoints were registered and resampled to a common unbiased within-participant reference using inverse-consistent registration, and the registered scans were subsequently processed with longitudinal SAMSEG. The cross-sectional SAMSEG model is applied to the within-participant template to initialize the longitudinal model, which then jointly estimates subject-specific and timepoint-specific parameters. Thus, the longitudinal SAMSEG estimates are derived from the longitudinal set of scans but do not use the separately generated cross-sectional SAMSEG segmentation outputs as inputs. GM and WM volume definitions were equivalent to those used for FreeSurfer.

#### FastSurfer

FastSurfer v1 consists of two main parts, FastSurferCNN and “recon-surf” (Henschel et al., 2020).

FastSurferCNN is a voxel-based deep learning architecture which segments the subcortical and cortical structures. The “recon-surf” surface-based module was used for cortical surface reconstruction and thickness analysis to correct the segmentations along the cortex and fine-tuned the volume estimates (e.g., surface-based partial volume correction). GM and WM volume definitions were equivalent to those used for FreeSurfer. Only cross-sectional FastSurfer processing was evaluated in this study; FastSurfer was therefore excluded from comparisons between cross-sectional and longitudinal processing.

#### SIENAX and SIENA-XL

The SIENAX method (Smith et al., 2002), implemented in FSL 6.0.4 (Jenkinson et al., 2012), is briefly explained here. The participant's brain and skull are extracted using Brain Extraction Tool (BET) and the brain is registered to a standard template for head-size normalization. Then tissue segmentation with partial volume estimation is applied. This yields normalized brain volume (NBV) split into gray and white matter. The pipeline-derived global GRAY and WHITE tissue volumes were used as the GM and WM measures, respectively. In the AD cohort, initial SIENAX processing with BET showed inadequate brain extraction in a subset of scans, resulting in over- or underestimation of volumetric measures. BET was therefore replaced by HD-BET for all AD scans to ensure a consistent brain-extraction procedure across the cohort (Isensee et al., 2019). The HD-BET-derived brain masks were subsequently dilated by a fixed number of voxels to retain sufficient surrounding head and skull signal for downstream SIENAX processing. The dilation extent was selected to improve brain extraction in problematic cases while minimizing its impact on the resulting volume estimates.

SIENA-XL method was used for longitudinal processing (Battaglini et al., 2018). SIENA-XL uses a newer brain extraction procedure and a multi-time-point intensity equalization step before performing the final segmentation, which also includes separate segmentation of deep GM structures. The SIENA-XL longitudinal workflow was applied directly to the longitudinal set of scans and did not use the separately generated SIENAX cross-sectional segmentation outputs as inputs. Accordingly, SIENA-XL processing of the AD cohort did not require the HD-BET-derived brain masks used for SIENAX.

#### CAT12

The CAT12 (Computational Anatomy Toolbox 12) version 2130 (CAT12.8.2) was used with default settings to extract GM and WM volumes (Gaser et al., 2024). Global GM and WM were obtained directly from the CAT12 whole-brain tissue-class volumes reported in the XML output. Hippocampal and thalamic volumes were obtained separately using the Neuromorphometrics atlas (Neuromorphometrics, Inc.neuromorphometrics.com) and were defined as the bilateral sum of GM, WM, and CSF tissue contributions within each region. These regional measures were therefore not treated as subsets of the CAT12 global GM volume. Longitudinal analysis creates an average image using symmetric rigid-body registration and bias field correction and segments it to produce a tissue probability map, which refines the segmentation of individual time points. Thus, the CAT12 longitudinal workflow uses the set of within-participant scans but does not use the separately generated cross-sectional CAT12 segmentation outputs as inputs. For the healthy control and MS cohort, the neuroplasticity model (optimized for small changes) was selected, while the aging model (optimized for large changes) was applied to the AD cohort. The former model averages time point-specific registrations to align segmentations in MNI space, while the latter adds smooth deformations to account for gradual brain changes, such as ventricular enlargement over time.

#### Intracranial volume normalization

Tissue volumes were reported as percentage of intracranial volume (% ICV). The longitudinal SAMSEG ICV estimates were constant across timepoints for each participant and used for normalization in all pipelines. This choice addresses the issues due to pipelines' differences in ICV estimation and ensures consistent scaling across all methods and timepoints (van Nederpelt et al., 2023). For longitudinal analyses, percentage change from baseline was calculated for each subject, tissue, pipeline, and processing mode as 100 × (*V*_follow − up_ – *V*_baseline_)/*V*_baseline_ where *V* denotes the normalized volume estimate.

### Statistical analyses

The statistical analyses were performed in R and RStudio (version 4.3.0, R Core Team, 2023, https://www.R-project.org/). These analyses quantified systematic differences, assessed volumetric agreement and repeatability between pipelines rather than absolute segmentation accuracy because of the lack of a reference standard.

#### Baseline systematic offset

To assess pipeline-dependent offsets in cross-sectional normalized volume estimates, baseline cross-sectional volumes were compared across pipelines separately for each cohort and tissue. Linear mixed-effects models were fitted with pipeline as a fixed effect and subject as a random intercept (*lme4* and *lmerTest* R package). The 12 *p*-values (three cohorts × four tissues) for the global pipeline effect were treated as one family and adjusted using the Benjamini–Hochberg procedure. For models with a significant false discovery rate (FDR)-adjusted global pipeline effect, pairwise *post hoc* comparisons were performed using estimated marginal means. Pairwise-comparison *p*-values were FDR-adjusted separately within each cohort and tissue. Statistical significance was defined as an FDR-adjusted *p*-value < 0.05.

#### Inter-pipeline agreement

Pairwise inter-pipeline agreement was quantified using intraclass correlation coefficients (ICCs). For each cohort and tissue, pairwise ICCs were computed between baseline normalized volume estimates from all available pipeline-mode outputs. Cross-sectional and longitudinal outputs were treated as separate implementations where applicable. ICCs were calculated using a two-way random-effects model for absolute agreement of single measurements (Shrout and Fleiss, 1979). The ICC ranges from 0 to 1 with higher values indicating stronger concordance between pipeline outputs, without implying anatomical accuracy.

Pairwise Pearson correlations were also computed as a complementary analysis to quantify linear association between pipeline outputs. Pearson correlation *p*-values were (FDR)-adjusted within each cohort and tissue using the Benjamini–Hochberg procedure (significance level: FDR-adjusted *p*-value < 0.05). Pearson correlations were not used as the primary agreement metric because correlation is insensitive to systematic offsets between methods.

#### Longitudinal change-from-baseline models

Longitudinal analyses focused primarily on change-from-baseline estimates rather than absolute volume over time, because baseline volumes differed systematically between pipelines. For each cohort, tissue, and processing mode, linear mixed-effects models were fitted to percentage change from baseline including Pipeline, Timepoint and their interaction as fixed effects, with subject as a random intercept. Pipeline main effect evaluated whether pipelines differed in average estimated change. The timepoint effect evaluated whether estimated change varied over follow-up. The pipeline-by-timepoint interaction evaluated whether pipelines produced different longitudinal trajectories. *P*-values were FDR-adjusted using the Benjamini–Hochberg procedure (significance level: FDR-adjusted *p*-value < 0.05) separately by term (pipeline/timepoint/pipeline-by-timepoint) across all cohort, tissue, and processing-mode models.

#### Within-pipeline stability and repeatability metrics

Within-pipeline stability was assessed in the healthy control cohort because this cohort was not expected to show substantial biological or pathology-related volume change over the short follow-up interval. For each pipeline, processing mode, and tissue, intra-pipeline ICCs were calculated across all available HC timepoints using normalized volume estimates. These ICCs provide an estimate of within-pipeline temporal consistency under short-interval conditions.

Additional repeatability metrics were computed in the HC cohort. For follow-up change-from-baseline estimates, we calculated the standard deviation (SD) of change, the mean absolute change, and the standard error of measurement (SEM) defined as SD change divided by √2.

Furthermore, a Bland–Altman repeatability coefficient (RC) was estimated. For the HC cohort, for each pipeline, tissue, and processing mode, a subject random-intercept model was fitted to the normalized volumes of all available tiempoints. The residual standard deviation from this model was interpreted as the within-subject standard deviation (*s*_w_). The RC was then calculated as 1.96 × √2 × *s*_w_. Lower values indicate lower within-subject measurement variability.

#### Effect of longitudinal processing

To assess whether longitudinal processing reduced variability relative to repeated cross-sectional processing, analyses were restricted to pipelines with both cross-sectional and longitudinal implementations. FastSurfer was excluded from this comparison. For HC and MS, within-subject variability was quantified as the standard deviation of percentage change-from-baseline estimates across follow-up visits for each participant, tissue, pipeline, and processing mode. The mean within-subject SD was then calculated across participants. The percentage reduction in variability with longitudinal processing was calculated as 100 × (SD_XSEC_ – SD_LONG_)/SD_XSEC_. Positive values indicate lower variability with longitudinal processing, whereas negative values indicate higher variability with longitudinal processing.

#### Sensitivity analyses of short-interval gray matter change in healthy controls

Because gray matter estimates in the HC cohort showed a common negative direction at follow-up, additional analyses were performed to assess whether this reflected a uniquely atypical baseline-to-first-follow-up interval or participant-level short-interval variability. Participant-level trajectories were visualized separately for each pipeline and processing mode, together with the mean and 95% confidence interval at each visit. To directly compare the first interval with subsequent intervals without treating multiple pipeline estimates from the same scans as independent observations, consecutive-visit percentage changes were first averaged across pipelines within each participant and interval. A linear mixed-effects model with Interval as a fixed effect and participant as a random intercept was then fitted separately for cross-sectional and longitudinal processing. A prespecified contrast compared the baseline-to-week-4 interval with the mean of the three subsequent intervals; additional pairwise contrasts compared it with each subsequent interval. To assess whether the main findings depended on the original baseline session, a separate sensitivity analysis used week 4 as the statistical reference and recalculated changes at weeks 8, 12, and 16. Bland–Altman analyses were additionally used to characterize signed baseline-to-follow-up differences and 95% limits of agreement.

#### Exploratory calibration analysis

To examine whether systematic differences between pipelines could be reduced through calibration, pairwise Deming regression models were fitted to ICV-normalized baseline volumes from the HC cohort. Models were estimated separately for each tissue, processing mode, and pipeline pair. For each pair, the measurements from one pipeline were transformed onto the scale of the other according to *Y* = *a* + *bX*, where *a* and *b* denote the HC-derived intercept and slope, respectively. Deming regression was used because both pipeline measurements were assumed to contain measurement error and neither pipeline represented an error-free reference standard. Deming regression was implemented in R using the closed-form estimator, assuming an error-variance ratio of 1. The model estimated the linear offset and scaling relationship required to transform one pipeline's measurements onto the scale of the other. The resulting coefficients were applied without refitting to baseline measurements from the MS and AD cohorts. Calibration performance was evaluated by comparing the absolute mean bias, mean absolute error (MAE), root-mean-square error (RMSE), and Lin's concordance correlation coefficient (CCC) before and after calibration. Because the HC calibration sample was small, these analyses were considered exploratory.

## Results

The analysis included three longitudinal cohorts: healthy controls, participants with relapsing-remitting MS, and participants with prodromal or mild AD. Figure 1 summarizes the cohort structure, preprocessing workflow, segmentation pipelines, and volumetric outcomes. The primary outputs were ICV-normalized gray matter, white matter, hippocampal, and thalamic volumes. Cross-sectional and longitudinal pipeline implementations were compared where available (FastSurfer v1 was evaluated only cross-sectionally).

**Figure 1:**
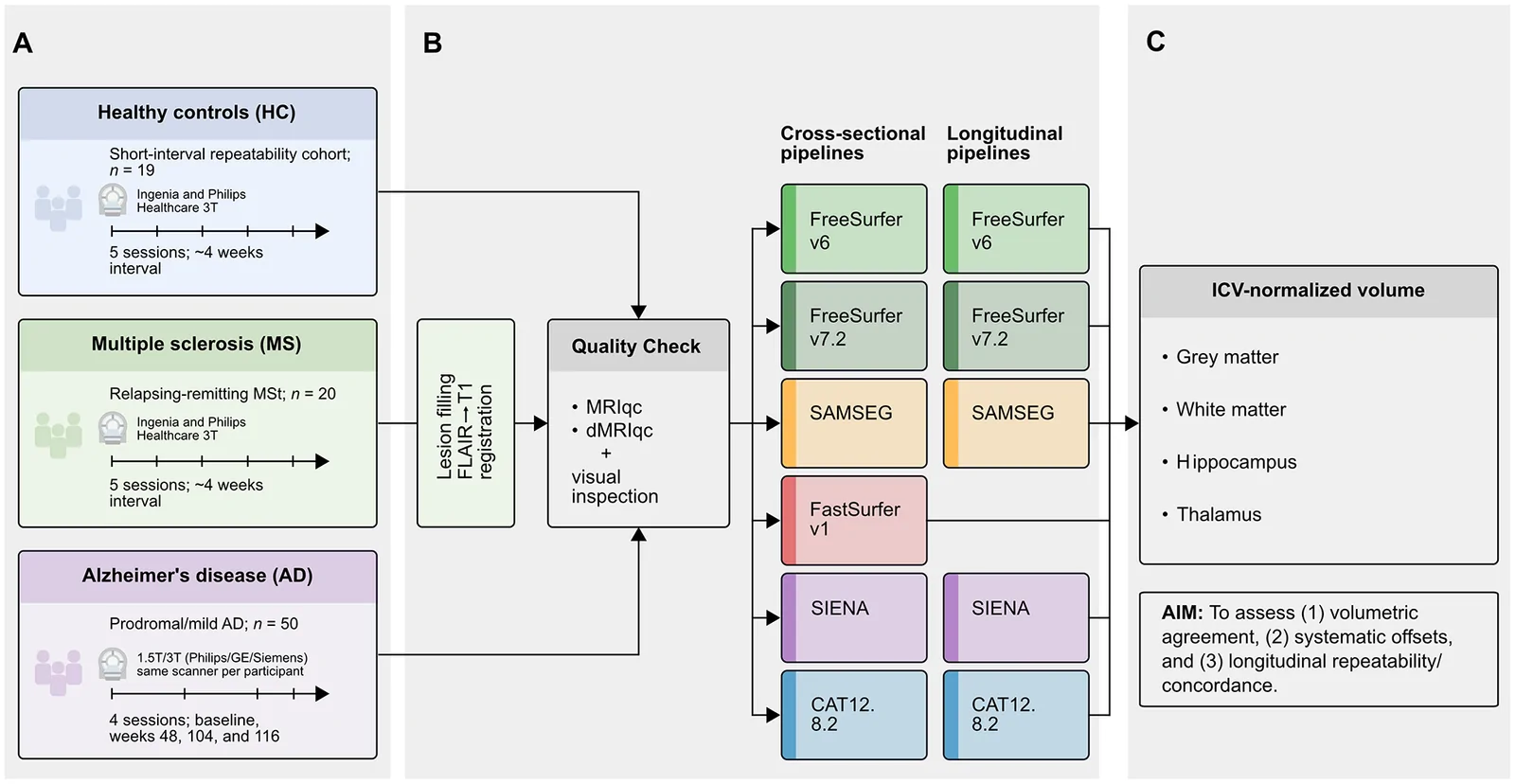
Study design and pipeline workflow. **(A)** Cohorts and MRI acquisition. **(B)** Image preprocessing and segmentation pipelines. **(C)** Volumetric outcomes.

The Section Results is organized according to the study aims: (1) systematic offsets and inter-pipeline agreement in baseline cross-sectional volumes; (2) longitudinal change-from-baseline patterns and within-pipeline stability; (3) the effect of longitudinal processing on variability. A summary of the main findings can be found in Table 4.

**Table 4:** Summary of observed systematic offsets and longitudinal change patterns across cohorts.

Tissue	Cohort	Relative baseline volume estimates	Observed change-from-baseline pattern
Gray matter	HC	CAT12 highest; FreeSurfer v6, FreeSurfer v7.2, SAMSEG, and FastSurfer intermediate; SIENAX lowest	Changes were small across pipelines, with no evidence of pipeline-specific trajectory shape
Gray matter	MS	CAT12 highest; FreeSurfer v6, FreeSurfer v7.2, SAMSEG, and FastSurfer intermediate; SIENAX lowest	Changes were broadly similar across pipelines
Gray matter	AD	CAT12 highest; FreeSurfer v6, FreeSurfer v7.2, SAMSEG, and FastSurfer intermediate; SIENAX lowest	Most pipelines estimated decline; SIENA-XL showed a smaller average change
White matter	HC	FreeSurfer and CAT12 toward higher estimates; FastSurfer and SAMSEG intermediate/lower; SIENAX among the lowest	Changes were generally similar across pipelines
White matter	MS	Baseline estimates were broadly similar; no significant overall pipeline effect after FDR correction	Changes were generally similar across pipelines
White matter	AD	SAMSEG highest; FreeSurfer v6/v7.2 intermediate; SIENAX and CAT12 lower	SAMSEG estimated more positive or less negative average change than most other pipelines
Hippocampus	HC	SAMSEG highest; FreeSurfer v6, FreeSurfer v7.2, and FastSurfer intermediate; CAT12 lowest	Changes were small across pipelines
Hippocampus	MS	SAMSEG highest; FreeSurfer v6, FreeSurfer v7.2, and FastSurfer intermediate; CAT12 lowest	Differences reflected average change magnitude rather than trajectory shape
Hippocampus	AD	SAMSEG highest; FreeSurfer v6, FreeSurfer v7.2, and FastSurfer intermediate; CAT12 lowest	SAMSEG differed from FreeSurfer/CAT12 in average change magnitude
Thalamus	HC	CAT12 highest; FreeSurfer v6, FreeSurfer v7.2, and FastSurfer intermediate; SAMSEG lowest	Changes were small across pipelines
Thalamus	MS	CAT12 highest; FreeSurfer v6, FreeSurfer v7.2, and FastSurfer intermediate; SAMSEG lowest	Limited differences in average change magnitude
Thalamus	AD	CAT12 highest; FreeSurfer v6, FreeSurfer v7.2, and FastSurfer intermediate; SAMSEG lowest	Pipeline-dependent differences in average change magnitude persisted

This table summarizes descriptive patterns in normalized volume estimates and change-from-baseline trajectories across pipelines. Relative baseline volume estimates are presented descriptively from higher to intermediate to lower estimates across pipelines. The final column highlights tissue- and cohort-specific patterns in estimated longitudinal change, including cases where one pipeline differed from the majority of other pipelines.

### Dataset and quality-control summary

At baseline, the analysis included 19 healthy controls, 20 participants with MS, and 50 participants from the GRADUATE AD cohort. For longitudinal analyses, 17 healthy controls were retained because two participants had only one follow-up scan. In the MS cohort, longitudinally 18 participants were retained because two participants had incomplete follow-up data. In the GRADUATE cohort, processing failures in FreeSurfer v7 and SAMSEG affected five participants; these participants were excluded from the longitudinal analyses, resulting in a final AD sample of 45 participants. A pipeline- and processing-mode-specific summary of processing failures is provided in Supplementary Table S1.

All participants included in the analysis had passed the input-image QC procedures described in the Section Methods, and no additional participants were excluded because of T1-weighted image quality. During initial SIENAX processing of the AD cohort, inadequate brain extraction affected 11 timepoints from five participants and resulted in over- or underestimation of brain volumes. These scans were reprocessed using the revised HD-BET-based brain-extraction procedure and were retained in the final analysis.

No manual corrections were performed, and no additional outputs were excluded after visual quality control.

### Baseline cross-sectional volume estimates showed systematic pipeline-dependent offsets

Baseline normalized volume estimates differed systematically across pipelines for all tissues and cohorts with the only exception of WM in MS (FDR-adjusted *p* = 0.36; all other FDR-adjusted *p* < 0.001; Figure 2; Table 5). These differences were most evident for gray matter and subcortical structures, whereas white matter estimates showed comparatively smaller offsets with no statistical difference in the MS cohort.

**Figure 2:**
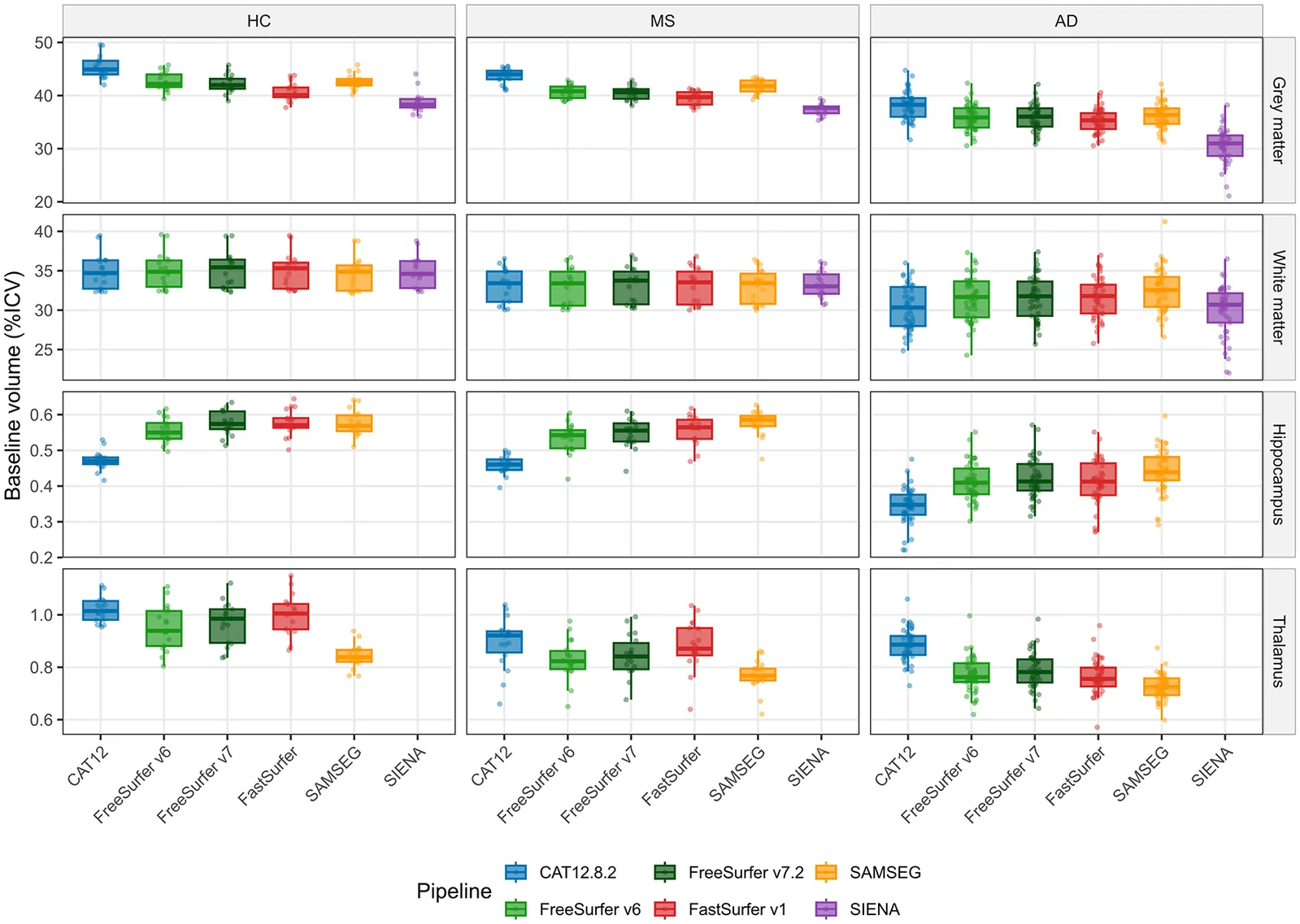
Cross-sectional normalized volume estimates differ systematically between segmentation pipelines. Baseline brain volume estimates are shown as percentage of intracranial volume (% ICV) for gray matter, white matter, hippocampus, and thalamus across healthy control (HC), multiple sclerosis (MS), and Alzheimer's disease (AD) cohorts. Each point represents one participant, and boxplots show the median and interquartile range for each pipeline. Volumes were normalized using longitudinal SAMSEG-derived ICV for all pipelines to ensure a common denominator across methods. This figure is intended to show systematic pipeline-dependent offsets in absolute volume estimates, rather than segmentation accuracy.

**Table 5:** Baseline volume model.

Cohort	Tissue	Term	*F* value	FDR-adjusted *p*-value
HC	Gray matter	Pipeline	328.45	< 0.001
HC	White matter	Pipeline	6.19	< 0.001
HC	Hippocampus	Pipeline	239.33	< 0.001
HC	Thalamus	Pipeline	116.51	< 0.001
MS	Gray matter	Pipeline	297.02	< 0.001
MS	White matter	Pipeline	1.11	0.36
MS	Hippocampus	Pipeline	187.60	< 0.001
MS	Thalamus	Pipeline	49.60	< 0.001
AD	Gray matter	Pipeline	309.76	< 0.001
AD	White matter	Pipeline	30.36	< 0.001
AD	Hippocampus	Pipeline	225.55	< 0.001
AD	Thalamus	Pipeline	320.30	< 0.001

This table reporting results from the linear mixed-effects models testing the effect of pipeline type on baseline cross-sectional normalized volume estimates. The results are separated by cohort and tissue. Models included pipeline as a fixed effect and subject as a random intercept. *P*-values were FDR-adjusted across all baseline pipeline tests.

For gray matter, CAT12.8.2 generally produced the highest normalized volume estimates, while SIENAX produced the lowest estimates. This pattern was observed across HC, MS, and AD cohorts, although the magnitude of the offset varied by cohort. The CAT12–SIENAX differences were 6.59 normalized-volume units (% ICV) in HC (95% CI: 6.25–6.93), 6.27 in MS (5.93–6.61), and 7.60 in AD (7.20–8.00), all FDR-adjusted *p* < 0.001. FreeSurfer v6, FreeSurfer v7.2, SAMSEG, and FastSurfer v1 generally produced intermediate estimates, with FreeSurfer and FastSurfer v1 showing similar magnitudes.

White matter estimates were more consistent across pipelines than gray matter estimates. Nevertheless, systematic offsets were still observed. In the HC cohort, the largest significant difference was between FreeSurfer v7.2 and SAMSEG (difference: 0.42%ICV; 95% CI: 0.25–0.59; FDR-adjusted *p* < 0.001). In MS, pairwise differences ranged from −0.27 to 0.23 % ICV, and none remained significant after FDR correction (all FDR-adjusted *p* ≥ 0.59). Larger differences were observed in AD, where SAMSEG produced higher estimates than SIENAX by 2.53%ICV (95% CI: 2.05–3.00; FDR-adjusted *p* < 0.001) and higher estimates than CAT12.8.2 by 2.18% ICV (95% CI: 1.71–2.66); both *p*-value and FDR-adjusted *p* < 0.001).

For hippocampal volume, SAMSEG generally produced the highest estimates and CAT12.8.2 consistently produced the lowest estimates. The largest pairwise offsets were 0.11% ICV between FastSurfer v1 and CAT12.8.2 in HC (95% CI: 0.10–0.11), 0.12% ICV between SAMSEG and CAT12.8.2 in MS (95% CI: 0.11–0.13), and 0.10% ICV between SAMSEG and CAT12.8.2 in AD (95% CI: 0.09–0.10), both *p*-value and FDR-adjusted *p* < 0.001. For the thalamus, CAT12.8.2 produced the highest estimates and SAMSEG the lowest, with CAT12–SAMSEG differences of 0.18% ICV in HC (95% CI: 0.16–0.20), 0.13 in MS (95% CI: 0.11–0.15), and 0.16 in AD (95% CI: 0.15–0.17), both *p*-value and FDR-adjusted *p* < 0.001. These findings indicate that pipeline-specific anatomical definitions and segmentation strategies produced systematic offsets that were not uniform across tissue classes.

### Inter-pipeline agreement varied by tissue and cohort

Pairwise ICCs showed that inter-pipeline agreement depended strongly on tissue type and cohort context (Figure 3; Supplementary Table S2). Agreement was generally higher for white matter than for gray matter and subcortical structures. Gray matter agreement varied substantially across pipeline pairs, particularly when SIENAX or CAT12.8.2 was compared with other pipelines.

**Figure 3:**
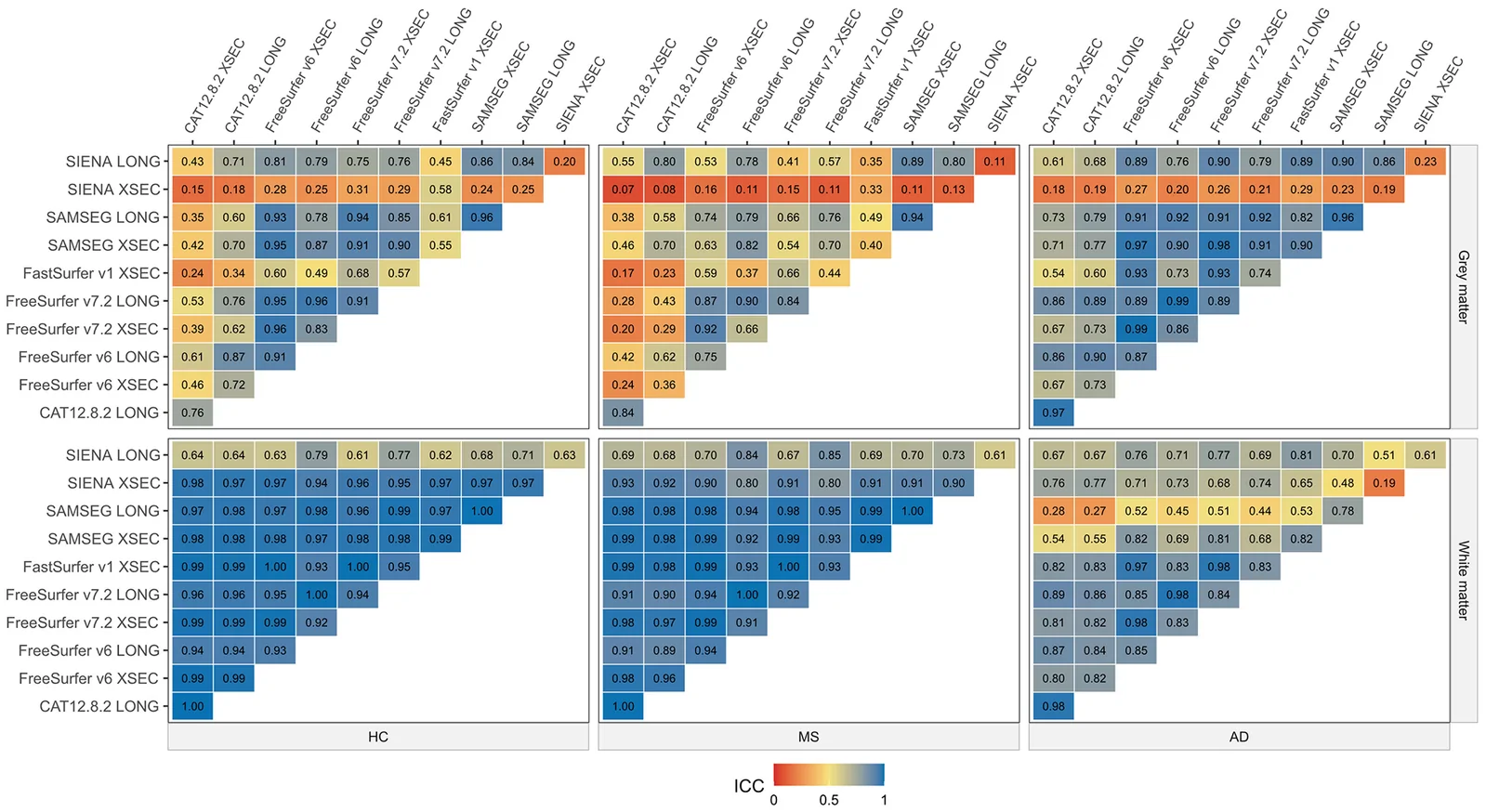
Inter-pipeline agreement of normalized volume estimates at baseline. Heatmaps show pairwise intraclass correlation coefficients (ICC) for absolute agreement between pipeline outputs at baseline, stratified by tissue and cohort. Cross-sectional and longitudinal implementations are displayed as separate pipeline-mode outputs where available. Higher ICC values are represented in blue and lower in red. Higher ICC indicates stronger concordance in normalized volume estimates between pipeline-mode combinations. ICC values should be interpreted as agreement between measurement methods.

White matter showed the strongest inter-pipeline concordance overall, especially in HC and MS. However, agreement was not uniformly high across all cohorts. In AD, WM agreement was reduced for some comparisons, particularly those involving SAMSEG, consistent with the divergent WM behavior observed in longitudinal analyses.

For gray matter, FreeSurfer v6, FreeSurfer v7.2, SAMSEG, and FastSurfer v1 showed relatively stronger agreement with each other than with SIENAX/-XL or CAT12 in several cohorts. FastSurfer v1 showed strong agreement with FreeSurfer, consistent with its FreeSurfer-derived training target. SIENAX showed lower gray matter agreement in HC and AD, indicating that SIENA-derived GM values should not be treated as interchangeable with other pipeline outputs.

Subcortical structures showed the greatest method dependence. Hippocampal agreement was strongest among FreeSurfer and FastSurfer v1 outputs, whereas CAT12.8.2 and SAMSEG differed more substantially from other methods. Thalamic agreement was also variable, with SAMSEG and CAT12.8.2 showing marked differences from FreeSurfer/FastSurfer in several comparisons. Additionally, Pearson correlation analyses showed broadly similar ranking patterns but were interpreted as secondary because high correlation can occur despite systematic offsets (Figure 4 and Supplementary Figure S1).

**Figure 4:**
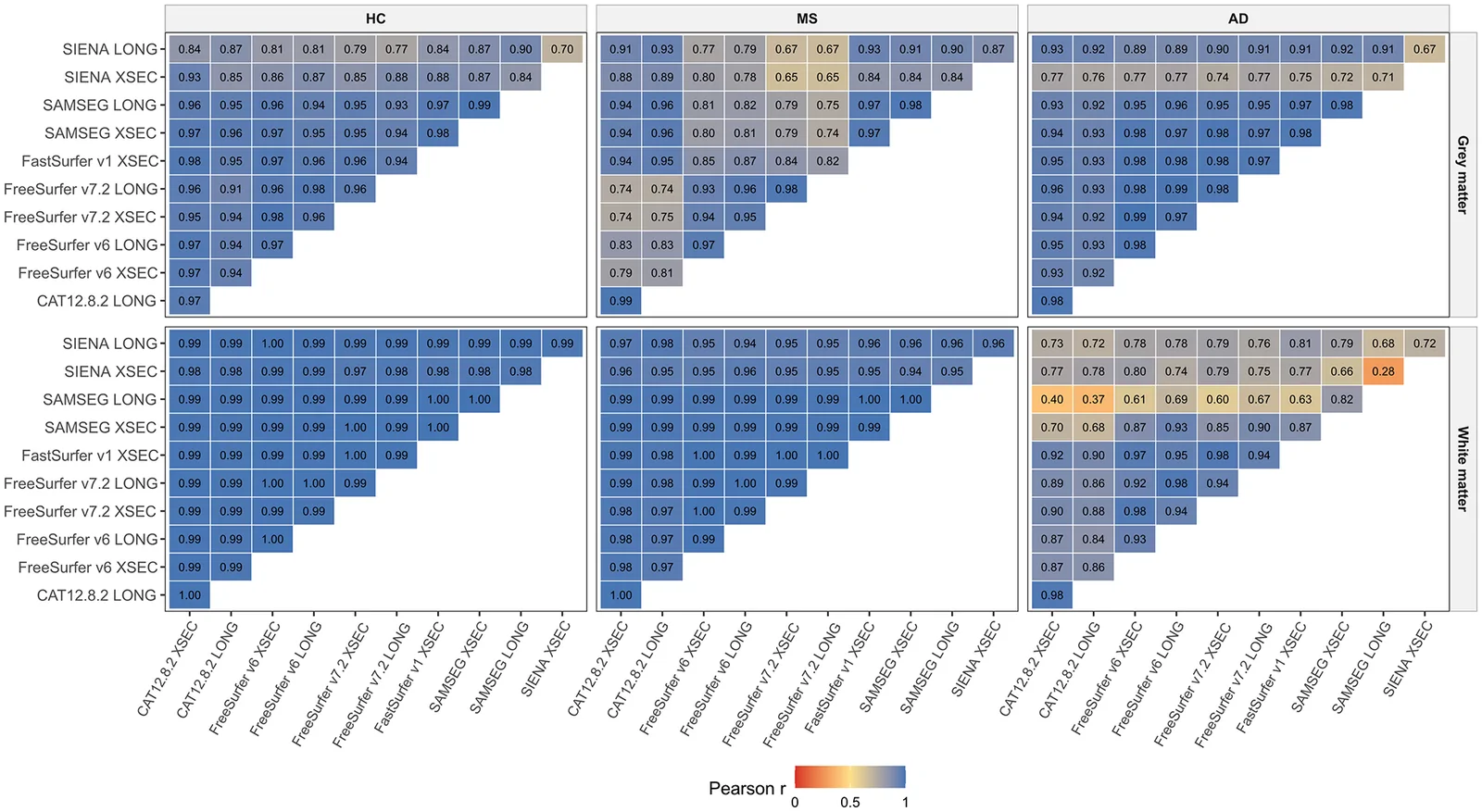
Pairwise Pearson correlations for gray and white matter volume estimates at baseline. Heatmaps show pairwise Pearson correlation coefficients between baseline normalized volume estimates from cross-sectional and longitudinal implementations of each pipeline, stratified by cohort and tissue. Values inside tiles indicate Pearson's *r*. The FDR-adjusted *p*-values were ≤0.001 for all pairwise comparisons except for WM (see Supplementary Table S5). Higher correlations indicate stronger linear association between pipeline-derived estimates but do not imply agreement in absolute values or segmentation accuracy.

### Within-pipeline stability was high in healthy controls but varied by tissue and pipeline

Intra-pipeline ICCs across all healthy-control timepoints were generally high, supporting good within-pipeline temporal stability over the short follow-up period (Supplementary Figure S2). WM showed the highest stability, with ICCs ranging from 0.982 (95% CI: 0.964–0.992) to 0.997 (95% CI: 0.995–0.999), whereas gray-matter ICCs ranged from 0.932 (95% CI: 0.871–0.971) to 0.974 (95% CI: 0.949–0.989). Subcortical ICCs were also generally high but showed greater pipeline- and processing-mode dependence. For FreeSurfer v7.2, hippocampal ICCs increased from 0.932 (95% CI: 0.871–0.971) with cross-sectional processing to 0.991 (95% CI: 0.982–0.996) with longitudinal processing, while thalamic ICCs increased from 0.941 (95% CI: 0.887–0.975) to 0.992 (95% CI: 0.984–0.997). A similar pattern was observed for FreeSurfer v6, with longitudinal processing yielding higher hippocampal and thalamic ICCs than repeated cross-sectional processing.

The HC repeatability metrics provided a complementary numerical summary of this stability (Supplementary Table S3). Bland–Altman repeatability coefficients (RCs) were calculated from within-subject residual variability across all HC timepoints. Lower values indicate smaller expected differences between repeated measurements from the same participant. RCs ranged from 0.75 to 1.22 %ICV for gray matter and from 0.32 to 0.77% ICV for white matter. Hippocampal RCs ranged from 0.007 to 0.026% ICV, while thalamic RCs ranged from 0.010 to 0.057% ICV. Longitudinal processing reduced RCs for subcortical estimates in several pipelines, particularly FreeSurfer. Hippocampal RCs decreased from 0.019 to 0.011 %ICV with FreeSurfer v6 and from 0.026 to 0.010 %ICV with FreeSurfer v7.2, while thalamic RCs decreased from 0.057 to 0.020% ICV and from 0.053 to 0.020 %ICV, respectively. However, this effect was not uniform; for CAT12.8.2, the thalamic RC increased from 0.025 to 0.038% ICV with longitudinal processing.

### Change-from-baseline estimates reduced baseline offsets but did not eliminate divergent longitudinal trajectories

Change-from-baseline trajectories are reported in Figure 5 for GM and WM and Supplementary Figure S4 for hippocampus and thalamus. Expressing follow-up measurements relative to each subject's own baseline reduced the influence of absolute baseline offsets between pipelines. In the relative percentage-change models, the pipeline-by-timepoint interaction was not significant for any tissue, cohort, or processing mode after FDR correction (interaction *F*-values range: 0.12–1.61; raw *p*-values range: 0.10–1.00; all FDR-adjusted *p* = 1.00), indicating no statistical evidence that pipelines differed in longitudinal trajectory shape after baseline normalization. The same conclusion was obtained when the models were repeated using signed change in ICV-normalized volume, for which all pipeline-by-timepoint interactions also remained nonsignificant (*F* = 0.10–1.50, raw *p* = 0.13–1.00; all FDR-adjusted *p* = 1.00; Supplementary Table S4).

**Figure 5:**
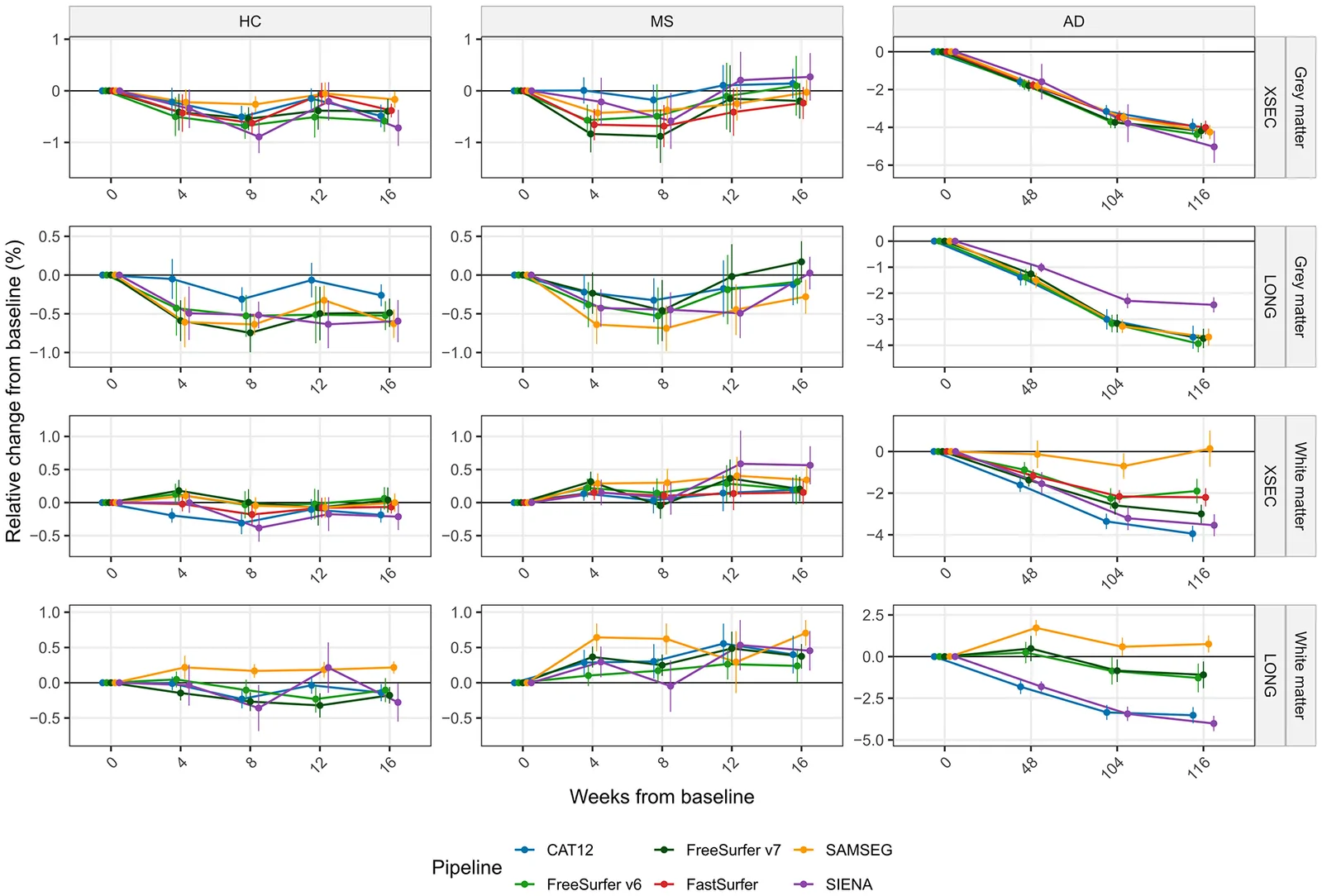
Longitudinal change-from-baseline estimates by segmentation pipeline. Mean percentage change from baseline is shown over time for each pipeline, stratified by tissue, processing mode, and cohort. Error bars represent the standard error of the mean across participants. The horizontal reference line indicates no change from baseline. This figure evaluates whether pipelines estimate similar longitudinal trajectories after removal of baseline offsets by expressing each subject's follow-up volumes relative to their own baseline value.

In HC, significant pipeline main effects in the relative-change models were observed for longitudinally processed white matter (*F* = 4.41, raw *p* = 0.002, FDR-adjusted *p* = 0.005) and cross-sectionally processed hippocampal estimates (*F* = 4.37, raw *p* = 0.002, FDR-adjusted *p* = 0.005). The cross-sectional white-matter pipeline effect did not survive FDR correction (*F* = 2.58, raw *p* = 0.03, FDR-adjusted *p* = 0.06), and no timepoint effect was significant after FDR correction. In MS, a significant pipeline effect was observed for cross-sectionally processed hippocampal estimates (*F* = 6.10, raw and FDR-adjusted *p* < 0.001). Significant timepoint effects were observed for cross-sectional GM (*F* = 4.89, raw *p* = 0.002, FDR-adjusted *p* = 0.006), longitudinal GM (*F* = 3.69, raw *p* = 0.01, FDR-adjusted *p* = 0.03), and cross-sectional hippocampal estimates (*F* = 4.01, raw *p* = 0.008, FDR-adjusted *p* = 0.02). The signed-change analyses showed the same overall pattern, with only minor shifts around the FDR significance threshold.

In AD, timepoint effects were significant across all tissues and processing modes, consistent with measurable longitudinal volume change. Significant pipeline main effects were also observed in seven of the eight models, with cross-sectional GM as the only exception, indicating differences in the average magnitude of estimated change across pipelines. This pattern was preserved in the signed-change analysis. However, no pipeline-by-timepoint interaction was significant after FDR correction, providing no evidence that these between-pipeline differences varied over time or represented different trajectory shapes. Most pipelines estimated progressive GM decline, although SIENA-XL showed a numerically smaller longitudinal GM change than FreeSurfer, SAMSEG, and CAT12.8.2. For WM, SAMSEG showed a visually divergent pattern, estimating positive or less negative change compared with the decline estimated by most other pipelines. For hippocampus, SAMSEG differed in average change magnitude from FreeSurfer/CAT12.8.2, while thalamic estimates showed persistent method-dependent differences, including higher CAT12.8.2 and lower SAMSEG estimates.

Overall, relative percentage-change and signed-change analyses produced concordant conclusions. Change-from-baseline analysis reduced the impact of baseline volume offsets and showed generally concordant trajectory shapes across pipelines. Nevertheless, significant pipeline main effects in selected tissues, particularly in AD and subcortical regions, showed that change estimates were not fully interchangeable across methods.

### Longitudinal processing reduced variability in selected settings but not uniformly

The effect of longitudinal processing on within-subject variability is summarized in Figure 6. This analysis was restricted to HC and MS cohorts and to pipelines with both cross-sectional and longitudinal processing modes (FastSurfer v1 was excluded).

**Figure 6:**
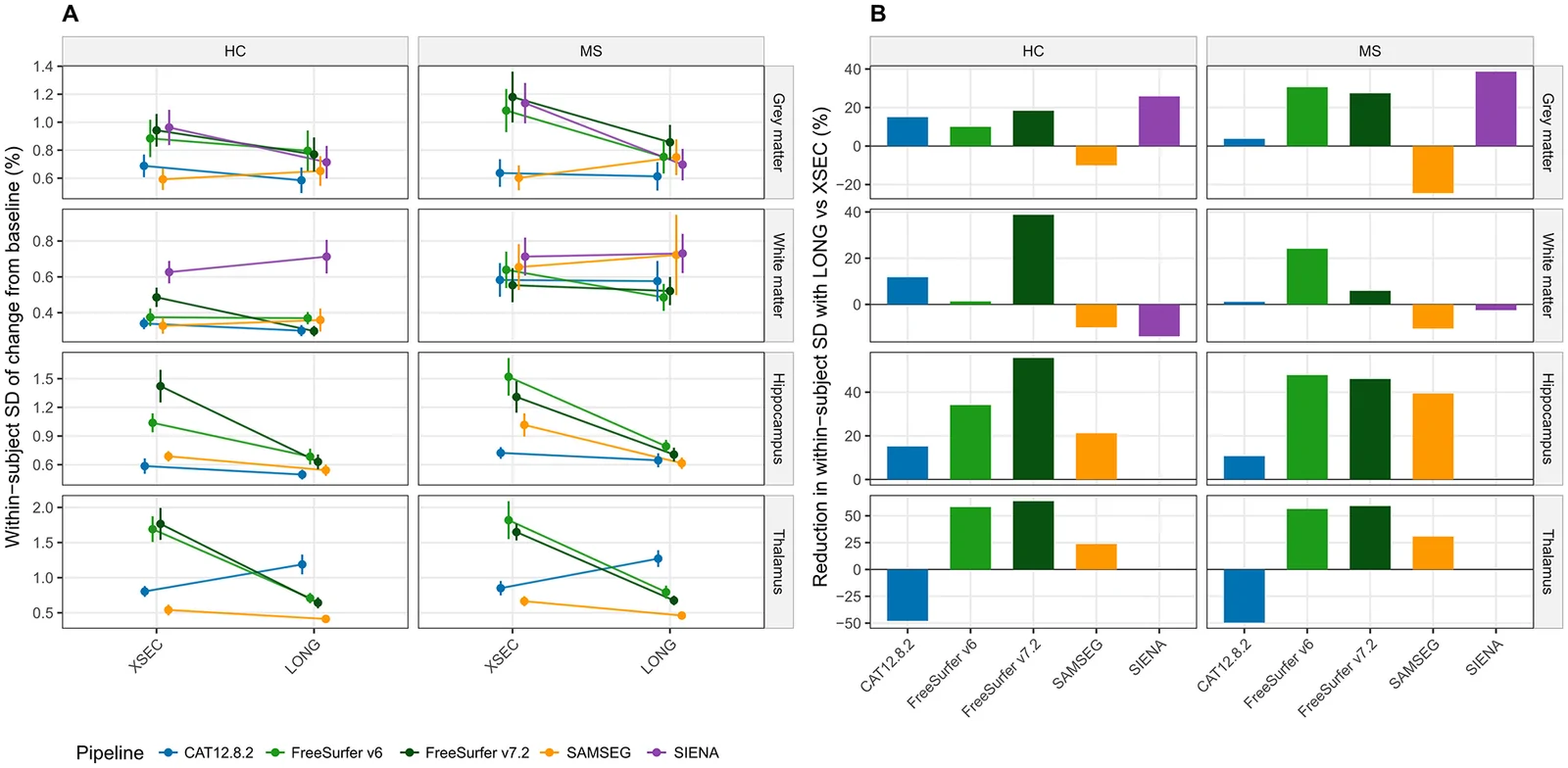
Effect of longitudinal processing on within-subject variability of volume change estimates. Panel **(A)** shows the mean within-subject standard deviation (SD) of percentage change-from-baseline estimates for repeated cross-sectional processing and longitudinal processing, stratified by tissue and cohort. Error bars represent the standard error of the mean across participants. Panel **(B)** shows the percentage reduction in within-subject variability with longitudinal processing, calculated as 100 × (SD_XSEC_ – SD_LONG_)/SD_XSEC_. Positive values indicate lower variability with longitudinal processing, whereas negative values indicate higher variability. Analyses are restricted to HC and MS cohorts, where short-interval biological change was expected to be small. For FreeSurfer, the longitudinal workflow uses information from the corresponding cross-sectional processing; therefore, the cross-sectional and longitudinal outputs are not independent. In contrast, the longitudinal implementations of SAMSEG and CAT12 use the within-participant set of scans rather than the separately generated cross-sectional segmentation outputs. FastSurfer was excluded because no longitudinal implementation was analyzed.

In HC, longitudinal processing reduced within-subject SD of change estimates for several tissue-pipeline combinations, particularly for FreeSurfer-derived hippocampal and thalamic estimates. For thalamic volume, within-subject SD decreased from 1.69 to 0.71% with FreeSurfer v6 and from 1.77 to 0.64% with FreeSurfer v7.2, corresponding to reductions of 58.1 and 63.6%, respectively. For hippocampal volume, SD decreased from 1.04 to 0.68% with FreeSurfer v6 and from 1.42 to 0.63% with FreeSurfer v7.2, corresponding to reductions of 34.1% and 55.7%. SAMSEG also reduced variability for the hippocampus and thalamus by 21.2% and 23.8%, respectively. This indicates improved short-interval stability for selected subcortical outputs when longitudinal processing was used. However, the effect was not uniform. Some pipelines showed little change, and in some cases variability increased under longitudinal processing. Namely, SAMSEG longitudinal showed increased variability by 10% and by 9.9% in gray and white matter, respectively. Additionally, SIENAX longitudinally reported an increased variability of 13.8% in white matter.

In MS, longitudinal processing also reduced variability for several FreeSurfer hippocampal and thalamic estimates. The benefit was less consistent for gray matter and white matter, where reductions varied by pipeline. Thalamic SD decreased from 1.82 to 0.79% with FreeSurfer v6 and from 1.65 to 0.68% with FreeSurfer v7.2, corresponding to reductions of 56.4% and 59.0%. Hippocampal variability decreased by 47.8% with FreeSurfer v6, 46.1% with FreeSurfer v7.2, 39.4% with SAMSEG, and 10.7% with CAT12.8.2. For gray matter, reductions were 38.7% with SIENA-XL, 30.6% with FreeSurfer v6, 27.4% with FreeSurfer v7.2, and 3.7% with CAT12.8.2, whereas SAMSEG increased variability by 24.5%. White matter reductions were smaller, reaching 24.1% with FreeSurfer v6, 5.9% with FreeSurfer v7.2, and 1.1% with CAT12.8.2; variability increased by 10.4% with SAMSEG and 2.5% with SIENA-XL. CAT12.8.2 also increased thalamic variability by 49.5%, from 0.85 to 1.27%.

Overall, the effect of longitudinal processing ranged from a 49.5% increase to a 63.6% reduction in within-subject variability. The largest and most consistent improvements were observed for FreeSurfer-derived hippocampal and thalamic measurements, whereas effects on gray and white matter were smaller and more pipeline-dependent. These findings demonstrate that longitudinal processing can improve the stability of selected volumetric outputs but should not be assumed to reduce variability uniformly across pipelines and tissues.

### Sensitivity analyses of short-interval gray matter change in healthy controls

Participant-level trajectories showed substantial short-interval variability in both directions, indicating that the negative mean change at the first follow-up was not a uniform participant-level effect (see Supplementary Figure S5). When consecutive-visit changes were averaged across pipelines within participants, the baseline-to-week-4 interval was not significantly different from the mean of the subsequent intervals for either cross-sectional processing (difference −0.385% ICV, 95% CI −1.19 to 0.42; *p* = 0.6) or longitudinal processing (−0.420 percentage points, 95% CI −1.14 to 0.30; *p* = 0.4). None of the comparisons between the baseline-to-week-4 interval and the individual subsequent intervals were statistically significant.

Re-referencing the analysis to week 4 similarly showed small subsequent changes, with all pipeline-specific 95% confidence intervals including zero comparison (Supplementary Table S6). Bland–Altman analyses showed small average signed differences relative to the substantially wider participant-level limits of agreement (Supplementary Table S6).

### Exploratory calibration analysis

HC-derived calibration reduced the offset/bias between pipelines, improving volume estimate agreement for many, but not all, cross-cohort comparisons. Specifically, across 164 transfers to the MS and AD cohorts, median absolute bias decreased from 0.22 to 0.11% ICV, median MAE decreased from 0.45 to 0.34% ICV, and median concordance increased from 0.71 to 0.82. Performance was strongly cohort dependent. When transferred to MS, calibration reduced MAE in 82.9% of comparisons and increased median concordance from 0.70 to 0.89. In contrast, transfer to AD was less consistent: MAE improved in 61% of comparisons, but median MAE increased from 0.73 to 0.86%ICV because some mappings produced substantial overcorrection. The better improvement reported in the MS cohort might be partially because HC and MS cohorts were closely matched for age and sex and were acquired using the same scanner and acquisition protocol. Calibration performance also varied by tissue and pipeline pair (see Supplementary Table S8).

## Discussion

This study employed a multi-cohort design to compare six commonly used brain segmentation pipelines across healthy-control, MS, and AD cohorts, with an emphasis on systematic offsets, inter-pipeline agreement, longitudinal change estimates, and repeatability. A key strength was the inclusion of a healthy control dataset with multiple MRI images acquired over a short period of time, establishing a baseline for assessing test-retest reliability while reducing the confounding factor of brain volume changes. Furthermore, the inclusion of MS and AD cohorts allowed the evaluation of pipeline performance under distinct pathological conditions, i.e., white matter lesions and brain volume loss.

The main findings were three-fold. First, pipelines produced systematic differences in baseline normalized volumes, particularly for gray matter and subcortical structures (hippocampus and thalamus). Second, inter-pipeline agreement was strongly tissue- and cohort-dependent: white matter estimates were generally more concordant, whereas gray matter, hippocampal, and thalamic estimates showed greater method dependence. Third, longitudinal processing reduced within-subject variability in selected settings, particularly for FreeSurfer-derived subcortical measures, but the benefit was not uniform across pipelines, tissues, or cohorts.

To summarize, the degree and type of disagreement between segmentation here pipelines depended on tissue, cohort, and processing mode, and longitudinal processing did not uniformly reduce variability.

### Systematic offsets indicate that absolute volumes are not directly comparable in native space

Substantial baseline offsets were observed across pipelines. Gray matter estimates were generally highest with CAT12 and lowest with SIENAX, while hippocampal and thalamic estimates showed strong pipeline-specific patterns, with SAMSEG producing higher hippocampal but lower thalamic estimates and CAT12 showing the opposite pattern. These differences were systematic, as demonstrated by significant pipeline effects in 11 of 12 cohort–tissue comparisons, and likely reflect differences in pipeline design, anatomical definitions, and intended applications.

FreeSurfer estimates anatomically labeled cortical and subcortical volumes using volumetric segmentation combined with cortical-surface reconstruction. FastSurfer uses a deep-learning segmentation model trained to reproduce FreeSurfer-style labels and subsequently applies a surface-reconstruction module. The comparatively strong agreement between these methods was therefore expected and is consistent with previous findings in healthy and clinical populations (Henschel et al., 2020). Previous work has also reported high concordance between these methods in both healthy and pathological groups, including AD, alongside improved test-retest repeatability (Henschel et al., 2020). Furthermore, models designed for early AD detection have exhibited comparable performance when trained on data processed by either pipelines (Bloch and Friedrich, 2021). Our study expands upon these findings by evaluating agreement with FreeSurfer v7.2 and by comparing FreeSurfer-based methods with pipelines that use substantially different segmentation frameworks.

SAMSEG uses a sequence-adaptive Bayesian generative model for voxel-based whole-brain segmentation, allowing image intensities and anatomical priors to be adapted to each scan. SIENAX was principally developed to estimate normalized global brain, gray matter, and white matter volumes using brain extraction, registration-based head-size normalization, and partial-volume tissue classification. Its outputs are therefore not based on the same cortical and regional anatomical definitions used by FreeSurfer-based approaches. SIENA-XL was designed primarily to measure longitudinal brain-volume change and additionally incorporates multi-timepoint intensity equalization and deep-gray matter segmentation. CAT12 uses tissue-probability modeling and spatial normalization, while hippocampal and thalamic estimates in the present study were derived from the Neuromorphometrics atlas.

Consequently, differences in tissue priors, partial-volume modeling, brain extraction, registration, cortical-boundary placement, and regional atlas definitions can produce pipeline-specific volume estimates even when the same image and a common ICV denominator are used. These methodological differences should also be interpreted in relation to the pipelines' original purposes. FreeSurfer and FastSurfer emphasize anatomically labeled cortical and subcortical morphometry; SAMSEG emphasizes robust and sequence-adaptive whole-brain segmentation; SIENAX and SIENA-XL were developed mainly for global tissue-volume and longitudinal atrophy estimation; and CAT12 is primarily a tissue-segmentation and voxel-based morphometry framework. Disagreement therefore does not necessarily indicate that one pipeline is erroneous. Rather, different pipelines may operationalize anatomical volume differently. In the absence of manual annotations or another external reference standard, the present study cannot determine which definition is anatomically most accurate.

### Exploratory calibration analysis

The exploratory calibration analysis showed that part of the systematic disagreement could be reduced using HC-derived Deming-regression equations. However, calibration transferred more successfully to the closely matched MS cohort than to the AD cohort and varied across tissues and pipeline pairs. The favorable HC-to-MS transfer likely reflects the cohort matching in terms of age, sex, scanner model and acquisition protocol. The less consistent transfer to AD indicates that calibration derived in one population cannot be assumed to generalize to a population with different demographic or disease-related characteristics. Calibration may therefore be useful in carefully matched settings, but a universal conversion table is unlikely to provide reliable harmonization across heterogeneous multicenter datasets. Any bridging analysis would need to represent the relevant sites, scanners, disease stages, and participant characteristics.

### Agreement was higher for white matter and lower for gray matter and subcortical structures

White matter estimates showed the strongest inter-pipeline agreement overall, consistent with prior work suggesting that global WM segmentation is relatively robust across methods, whereas gray matter and deep gray matter structures are more method-dependent (van Nederpelt et al., 2023; Kondrateva et al., 2025). However, the WM agreement was not uniformly high. In the AD cohort, SAMSEG showed a divergent WM pattern, estimating positive or less negative change where most other pipelines estimated decline.

Gray matter agreement was more variable. SIENAX showed lower agreement for GM in HC and AD, consistent with previous reports that SIENAX can be less reliable than cross-sectional FreeSurfer v6 for some volumetric measures and less precise for longitudinal change estimation (Durand-Dubief et al., 2012; Guo et al., 2019). Although SIENAX remains widely used for cross-sectional brain volume estimation (Steenwijk et al., 2017; van Nederpelt et al., 2023), our results suggest that SIENAX-derived GM estimates should be interpreted cautiously when compared with other pipeline outputs. Conversely, SIENA-XL achieved high reliability in healthy controls and stronger alignment in AD, confirming its higher measurement sensitivity (Battaglini et al., 2018).

In the MS cohort, lower agreement for GM may partly reflect the effect of white matter lesions on tissue classification, despite lesion filling. Lesion filling and pathology-related intensity abnormalities might affect global intensity normalization, partial-volume classification, and cortical-boundary placement differently across pipelines, particularly near the gray–white interface. This is consistent with previous work showing that lesions can influence brain volumetry and that lesion processing choices can affect GM and WM estimates in MS (van Nederpelt et al., 2023).

### Longitudinal change estimates reduced but did not eliminate pipeline differences

Expressing follow-up values as change from baseline reduced the influence of absolute volume offsets. In HC and MS, estimated changes were generally small, supporting the interpretation that short-interval fluctuations mostly reflect measurement variability rather than substantial biological change. In AD, most pipelines estimated progressive gray matter decline, but SIENA-XL estimated slower GM change than several other methods.

These findings indicate that cross-sectional agreement does not guarantee concordant longitudinal trajectories. This was particularly evident for AD white matter, where SAMSEG estimated positive or less negative change compared with the majority of other pipelines. Therefore, longitudinal studies should not assume that change-from-baseline fully resolves pipeline differences.

### Longitudinal processing improved stability in selected settings

Longitudinal processing reduced within-subject variability in selected tissue-pipeline combinations, most clearly for FreeSurfer-derived hippocampal and thalamic estimates. This is consistent with the rationale for longitudinal processing, where within-subject templates can reduce variability introduced by independent segmentation of each timepoint.

The subcortical findings are broadly consistent with prior work suggesting that SAMSEG and longitudinal processing approaches can improve reliability for subcortical structures and disease-related change estimation (Sederevičius et al., 2021). In our data, longitudinal FreeSurfer versions appeared more stable than cross-sectional FreeSurfer for hippocampus and thalamus. However, SAMSEG and CAT12 still differed from other pipelines in absolute subcortical volume estimates and, in some cohorts, in change-from-baseline trajectories. CAT12 subcortical differences may partly reflect its use of the Neuromorphometrics atlas for ROI extraction. Previous work has suggested in a head-to-head comparison with FreeSurfer v7.1 that SAMSEG may be preferable for longitudinal subcortical research involving disease progression (Sederevičius et al., 2021) because of comparable reliability but tighter test-retest metrics, lower volume differences and improved spatial overlap relative to FreeSurfer. It also avoided early-attribution hippocampal decline seen in FreeSurfer v7.1 and more sensitively detected volume loss in aging and AD cohorts (Sederevičius et al., 2021). Our findings showed that SAMSEG consistently differed from other pipelines in MS and AD cohorts.

Overall, longitudinal processing should not be described as uniformly superior. Its benefit was tissue- and pipeline-dependent and should be evaluated for the specific outcome and cohort under study.

### Practical implications for longitudinal neuroimaging studies

The main practical implication is that pipeline choice should remain consistent within a study. Outputs from different pipelines should not be treated as interchangeable on their native scales without prior agreement assessment or harmonization, particularly for gray matter, hippocampus, and thalamus. For white matter, inter-pipeline agreement was generally stronger, but AD results showed that even WM estimates can diverge depending on pipeline and cohort context.

Consistency is also required across software versions within the same pipeline family. FreeSurfer v6 and v7 showed relatively strong agreement and generally smaller differences than comparisons between methodologically distinct pipelines, but their outputs were not identical. Software-version compatibility should therefore not be assumed. Changing FreeSurfer versions during follow-up may introduce a measurement shift that is difficult to distinguish from biological change, especially for cortical gray matter, hippocampal, and thalamic outcomes, where small methodological differences may be comparable to the changes expected over short follow-up periods.

All timepoints from a participant should preferably be processed using the same FreeSurfer version, processing stream, command options, and computational environment. For existing longitudinal datasets containing outputs from multiple versions, the preferred approach is to reprocess all scans using one prespecified version and, when the longitudinal stream is used, to recreate the within-subject templates from the complete set of timepoints. Combining v6 and v7 outputs without reprocessing should be considered only after a bridging analysis in which the same representative scans are processed with both versions to quantify tissue- and cohort-specific bias, participant-level agreement, and longitudinal repeatability. Any resulting calibration should be supported by sensitivity analyses, and the software version and processing configuration should be reported explicitly.

For longitudinal trials, change-from-baseline estimates can reduce baseline offsets but do not fully eliminate pipeline-dependent differences in trajectories. Therefore, studies should predefine the pipeline, version, processing mode, ICV normalization strategy, lesion-processing approach, and quality-control criteria. Comparisons across studies should consider these methodological details before interpreting differences as biological.

### MS-specific considerations

The MS cohort required lesion filling before segmentation, which is an important methodological step because lesions can affect tissue classification and brain volume estimation (Guo et al., 2019; van Nederpelt et al., 2023). However, lesion filling also introduces a pipeline dependency. Differences between pipelines in MS may therefore reflect not only segmentation algorithms but also how each method responds to lesion-filled T1-weighted images (Guo et al., 2019; van Nederpelt et al., 2023).

Because MS can show short-interval changes related to inflammation, lesion activity, relapse status, hydration, or pseudoatrophy-like effects, repeatability metrics were emphasized primarily in the HC cohort. MS short-interval variability remains informative, but it cannot be interpreted as pure measurement error.

### Sensitivity analyses of gray matter change in healthy controls

The sensitivity analyses performed on the HC cohort suggest that the negative mean change relative to the baseline should be interpreted cautiously, but do not provide evidence that the baseline represents an invalid timepoint. The participant-level variability and the absence of a uniquely abnormal baseline-to-first-follow-up interval indicate that the observed group-level shift falls within the broader short-interval variability of the cohort. Moreover, no independent acquisition, image-quality, or segmentation failure was identified, we therefore retained the prespecified baseline in the primary analysis. Potential physiological, acquisition, or session-related sources of this variability are considered in the Limitations.

### Limitations and future work

This study has several limitations that should be acknowledged. First, the sample size for the healthy control and MS cohorts were relatively small which may reduce statistical power. In addition, although HC participants were scanned at approximately the same time of day across visits, with a tolerated variation of 2–3 h, residual diurnal or session-related effects cannot be completely excluded. Potential shared sources of short-interval variability include hydration and other internal physiological states, head positioning, motion, intensity inhomogeneity, and short-term variation in scanner state. Second, both datasets originated from a single site, lacking scanner variability, potentially constraining generalizability. Third, the absence of a ground truth restricted our analysis to relative comparisons, precluding the assessment of absolute segmentation accuracy. For MS, results were likely influenced by the specific lesion-filling strategy employed, and more modern lesion-filling approaches were not evaluated. In AD, white matter hyperintensities (WMHs) may affect T1-based segmentation and introduce systematic bias in volumetric estimates; such effects have been reported for FreeSurfer v6 (Dadar et al., 2021). In the present study, WMH burden could not be quantified because FLAIR imaging was unavailable in the AD cohort, precluding direct assessment of the potential contribution of WMHs to the observed volumetric differences and longitudinal changes. Newer versions of several evaluated pipelines were not included in this comparison. Furthermore, longitudinal analysis did not include FastSurfer. Additionally, we did not compare segmentation outputs to manual annotations (the “gold standard”), a common limitation in the field due to the scarcity of such datasets for AD and MS (Valverde et al., 2015).

Future research should prioritize expanding dataset diversity by incorporating larger, multi-site cohorts with heterogeneous scanning parameters. Investigating the impact of different lesion filling techniques could strengthen results in MS. Future work should extend the present comparison by including newer versions of the evaluated pipelines to examine whether the observed patterns are preserved across software versions. Furthermore, evaluating longitudinal FastSurfer and the Clinical FreeSurfer (Gopinath et al., 2024) pipeline alongside standard versions would provide a more exhaustive overview. Finally, benchmarking additional pipelines commonly used in trials (e.g., SPM, FSL, SynthSeg) across a broader range of brain structures would enable a more comprehensive assessment of segmentation reliability.

## Conclusion

This study shows that volumetric outputs from common brain segmentation pipelines should not be assumed to be directly interchangeable across pipelines or software versions on their native scales. Baseline offsets were substantial for gray matter and subcortical structures, while white matter estimates showed comparatively stronger agreement. Change-from-baseline analyses reduced baseline offsets and produced broadly similar trajectory shapes, but pipeline-dependent differences in the magnitude of estimated change remained in selected tissues and cohorts like the AD. Longitudinal processing improved stability in selected settings, especially for some subcortical outputs, but did not provide uniform improvement across all pipelines or tissues. Some differences may be reduced through validated calibration, particularly under closely matched conditions, but consistent processing with the same pipeline, version, and configuration remains the most robust approach for longitudinal studies and clinical trials.

## Data Availability

The data analyzed in this study is subject to the following licenses/restrictions: the Sherbrooke datasets are available upon agreement with the data owner. The graduate dataset requires a detailed, hypothesis-driven statistical analysis plan that is collaboratively developed by the requestor and company subject matter experts. Only derivatives data will be shared in agreement with the privacy law of the specific country. Anonymized records for individual patients across more than one data source external to Roche cannot, and should not, be linked due to a potential increase in risk of patient re-identification. Requests to access these datasets should be directed to Maxime.Descoteaux@USherbrooke.ca for the Sherbrooke dataset; glofct-nrd_biomarkers_imag@msxdl.roche.com for the Graduate dataset.

## References

[B1] Bateman R. J. Smith J. Donohue M. C. Delmar P. Abbas R. Salloway S. . (2023). Two phase 3 trials of gantenerumab in early Alzheimer's disease. N Engl J Med. 389, 1862–1876. doi: 10.1056/NEJMoa2304430 37966285 PMC10794000

[B2] Battaglini M. Jenkinson M. De Stefano N. (2012). Evaluating and reducing the impact of white matter lesions on brain volume measurements. Hum Brain Mapp. 33, 2062–2071. doi: 10.1002/hbm.21344 21882300 PMC6870255

[B3] Battaglini M. Jenkinson M. De Stefano N. (2018). SIENA-XL for improving the assessment of gray and white matter volume changes on brain MRI. Hum Brain Mapp. 39, 1063–1077. doi: 10.1002/hbm.23828 29222814 PMC6866496

[B4] Bigler E. D. Skiles M. Wade B. S. C. Abildskov T. J. Tustison N. J. Scheibel R. S. . (2020). FreeSurfer 5.3 versus 6.0: are volumes comparable? a chronic effects of neurotrauma consortium study. Brain Imaging Behav. 14, 1318–1327. doi: 10.1007/s11682-018-9994-x 30511116

[B5] Bittner T. Tonietto M. Klein G. Belusov A. Illiano V. Voyle N. . (2025). Biomarker treatment effects in two phase 3 trials of gantenerumab. Alzheimers Dement. J Alzheimers Assoc. 21:e14414. doi: 10.1002/alz.14414 39887500 PMC11848197

[B6] Bloch L. Friedrich C. M. (2021). “Comparison of automated volume extraction with FreeSurfer and FastSurfer for early Alzheimer's disease detection with machine learning,” in 2021 IEEE 34th international symposium on computer-based medical systems (CBMS). IEEE. doi: 10.1109/CBMS52027.2021.00096

[B7] Cerri S. Greve D. N. Hoopes A. Lundell H. Siebner H. R. Mühlau M. . (2023). An open-source tool for longitudinal whole-brain and white matter lesion segmentation. NeuroImage Clin. 38:103354. doi: 10.1016/j.nicl.2023.103354 36907041 PMC10024238

[B8] Dadar M. Potvin O. Camicioli R. Duchesne S. (2021). Beware of white matter hyperintensities causing systematic errors in FreeSurfer gray matter segmentations! Hum. Brain Mapp. 42, 2734–2745. doi: 10.1002/hbm.25398 PMC8127151 33783933

[B9] Durand-Dubief F. Belaroussi B. Armspach J. P. Dufour M. Roggerone S. Vukusic S. . (2012). Reliability of longitudinal brain volume loss measurements between 2 sites in patients with multiple sclerosis: comparison of 7 quantification techniques. AJNR Am. J. Neuroradiol. 33, 1918–1924. doi: 10.3174/ajnr.A3107 PMC7964600 22790248

[B10] Edde M. Houde F. Theaud G. Dumont M. Gilbert G. Houde J. C. . (2023a). Impact of follow ups, time interval and study duration in diffusion and myelin MRI clinical study in MS. NeuroImage Clin. 40:103529. doi: 10.1016/j.nicl.2023.103529 37857232 PMC10591008

[B11] Edde M. Theaud G. Dumont M. Théberge A. Valcourt-Caron A. Gilbert G. . (2023b). High-frequency longitudinal white matter diffusion- and myelin-based MRI database: Reliability and variability. Hum. Brain Mapp. 44, 3758–3780. doi: 10.1002/hbm.26310 37067096 PMC10203793

[B12] Filip P. Bednarik P. Eberly L. E. Moheet A. Svatkova A. Grohn H. . (2022). Different FreeSurfer versions might generate different statistical outcomes in case-control comparison studies. Neuroradiology 64, 765–773. doi: 10.1007/s00234-021-02862-0 34988592 PMC8916973

[B13] Fischl B. (2012). FreeSurfer. NeuroImage, 62. 774–781. doi: 10.1016/j.neuroimage.2012.01.021 22248573 PMC3685476

[B14] Fischl B. Salat D. H. Busa E. Albert M. Dieterich M. Haselgrove C. . (2002). Whole brain segmentation: automated labeling of neuroanatomical structures in the human brain. Neuron 33, 341–355. doi: 10.1016/S0896-6273(02)00569-X 11832223

[B15] Fischl B. van der Kouwe A. Destrieux C. Halgren E. Ségonne F. Salat D. H. . (2004). Automatically parcellating the human cerebral cortex. Cereb. Cortex 14, 11–22. doi: 10.1093/cercor/bhg087 14654453

[B16] Gaser C. Dahnke R. Thompson P. M. Kurth F. Luders E. The Alzheimer's Disease Neuroimaging Initiative (2024). CAT: a computational anatomy toolbox for the analysis of structural MRI data. GigaScience 13:giae049. doi: 10.1093/gigascience/giae049 PMC11299546 39102518

[B17] Glatard T. Lewis L. B. Ferreira da Silva R. Adalat R. Beck N. Lepage C. . (2015). Reproducibility of neuroimaging analyses across operating systems. Front. Neuroinform. 9:12. doi: 10.3389/fninf.2015.00012 PMC4408913 25964757

[B18] Gopinath K. Greve D. N. Magdamo C. Arnold S. Das S. Puonti O. . (2024). Recon-all-clinical: cortical surface reconstruction and analysis of heterogeneous clinical brain MRI. arXiv [Preprint]. arXiv:2409.03889. doi: 10.48550/arXiv.2409.03889 PMC12124945 40300378

[B19] Goto M. Abe O. Hagiwara A. Fujita S. Kamagata K. Hori M. . (2022). Advantages of using both voxel- and surface-based morphometry in cortical morphology analysis: a review of various applications. Magn. Reson. Med. Sci. 21, 41–57. doi: 10.2463/mrms.rev.2021-0096 35185061 PMC9199978

[B20] Guo C. Ferreira D. Fink K. Westman E. Granberg T. (2019). Repeatability and reproducibility of FreeSurfer, FSL-SIENAX and SPM brain volumetric measurements and the effect of lesion filling in multiple sclerosis. Eur. Radiol. 29, 1355–1364. doi: 10.1007/s00330-018-5710-x 30242503 PMC6510869

[B21] Haddad E. Pizzagalli F. Zhu A. H. Bhatt R. R. Islam T. Ba Gari I. . (2023). Multisite test-retest reliability and compatibility of brain metrics derived from FreeSurfer versions 7.1, 6.0, and 5.3. Hum. Brain Mapp. 44, 1515–1532. doi: 10.1002/hbm.26147 PMC9921222 36437735

[B22] Hedges E. P. Dimitrov M. Zahid U. Brito Vega B. Si S. Dickson H. . (2022). Reliability of structural MRI measurements: the effects of scan session, head tilt, inter-scan interval, acquisition sequence, FreeSurfer version and processing stream. NeuroImage 246:118751. doi: 10.1016/j.neuroimage.2021.118751 34848299 PMC8784825

[B23] Henschel L. Conjeti S. Estrada S. Diers K. Fischl B. Reuter M. (2020). FastSurfer - a fast and accurate deep learning based neuroimaging pipeline. NeuroImage 219:117012. doi: 10.1016/j.neuroimage.2020.117012 32526386 PMC7898243

[B24] Isensee F. Schell M. Pflueger I. Brugnara G. Bonekamp D. Neuberger U. . (2019). Automated brain extraction of multisequence MRI using artificial neural networks. Hum. Brain Mapp. 40, 4952–4964. doi: 10.1002/hbm.24750 PMC6865732 31403237

[B25] Jenkinson M. Beckmann CF. Behrens T. E. Woolrich M. W. Smith S.M. (2012). FSL. NeuroImage 62, 782–790. doi: 10.1016/j.neuroimage.2011.09.015 21979382

[B26] Knights H. Coleman A. Hobbs N. Z. Tabrizi S. J. Scahill R. I. (2024). Freesurfer software update significantly impacts striatal volumes in the Huntington's disease young adult study and will influence HD-ISS staging. J. Huntingtons Dis. 13, 77–90. doi: 10.3233/JHD-231512 38489194

[B27] Kondrateva E. Barg S. Vasiliev M. (2025). Benchmarking the reproducibility of brain MRI segmentation across scanners and time. arXiv [Preprint]. arXiv:2504.15931. doi: 10.48550/arXiv.2504.15931

[B28] Magon S. Gaetano L. Chakravarty M. M. Lerch J. P. Naegelin Y. Stippich C. . (2014). White matter lesion filling improves the accuracy of cortical thickness measurements in multiple sclerosis patients: a longitudinal study. BMC Neurosci. 15:106. doi: 10.1186/1471-2202-15-106 25200127 PMC4164794

[B29] Puonti O. Iglesias J. E. Van Leemput K. (2016). Fast and sequence-adaptive whole-brain segmentation using parametric Bayesian modeling. NeuroImage, 143. 235–249. doi: 10.1016/j.neuroimage.2016.09.011 PMC8117726 27612647

[B30] R Core Team (2023). R: A Language and Environment for Statistical Computing. Vienna: R Foundation for Statistical Computing. Available online at: https://www.R-project.org/

[B31] Reuter M. Schmansky N. J. Rosas H. D. Fischl B. (2012). Within-subject template estimation for unbiased longitudinal image analysis. NeuroImage 61, 1402–1418. doi: 10.1016/j.neuroimage.2012.02.084 22430496 PMC3389460

[B32] Sederevičius D. Vidal-Piñeiro D. Sørensen Ø. van Leemput K. Iglesias J. E. Dalca A. V. . (2021). Reliability and sensitivity of two whole-brain segmentation approaches included in FreeSurfer - ASEG and SAMSEG. NeuroImage 237:118113. doi: 10.1016/j.neuroimage.2021.118113 33940143 PMC9052126

[B33] Shrout P. E. Fleiss J. L. (1979). Intraclass correlations: uses in assessing rater reliability. Psychol. Bull. Hosp. Jt. Dis. 86, 420–428. doi: 10.1037/0033-2909.86.2.420 18839484

[B34] Singh M. K. (2023). Reproducibility and reliability of computing models in segmentation and volumetric measurement of brain. Ann. Neurosci. 30, 224–229. doi: 10.1177/09727531231159959 PMC10662274 38020401

[B35] Smith S. M. Zhang Y. Jenkinson M. Chen J. Matthews P. M. Federico A. . (2002). Accurate, robust, and automated longitudinal and cross-sectional brain change analysis. NeuroImage 17, 479–489. doi: 10.1006/nimg.2002.1040 12482100

[B36] Steenwijk M. D. Amiri H. Schoonheim M. M. de Sitter A. Barkhof F. Pouwels P. J. W. . (2017). Agreement of MSmetrix with established methods for measuring cross-sectional and longitudinal brain atrophy. NeuroImage Clin. 15, 843–853. doi: 10.1016/j.nicl.2017.06.034 28794970 PMC5540882

[B37] Valverde S. Oliver A. Cabezas M. Roura E. Lladó X. (2015). Comparison of 10 brain tissue segmentation methods using revisited IBSR annotations. J. Magn. Reson. Imaging 41, 93–101. doi: 10.1002/jmri.24517 24459099

[B38] van Nederpelt D. R. Amiri H. Brouwer I. Noteboom S. Mokkink L. B. Barkhof F. . (2023). Reliability of brain atrophy measurements in multiple sclerosis using MRI: an assessment of six freely available software packages for cross-sectional analyses. Neuroradiology 65, 1459–1472. doi: 10.1007/s00234-023-03189-8 37526657 PMC10497452

[B39] Zhang Y. Brady M. Smith S. (2001). Segmentation of brain MR images through a hidden Markov random field model and the expectation-maximization algorithm. IEEE Trans. Med. Imaging 20, 45–57. doi: 10.1109/42.906424 11293691

