# Supplementary material for: Agreement and longitudinal repeatability of established brain volumetry pipelines across healthy controls, multiple sclerosis, and Alzheimer's disease
Source: Front Neurosci. 2026 Oct 16;20:1917471. doi: 10.3389/fnins.2026.1917471 (PMC13720710; doi:10.3389/fnins.2026.1917471)
Supplement: Supplementary file 1 [file Data_Sheet_1.docx]

# Tables

| **QC stage** | **Cohort** | **Mode** | **Pipeline /**  **QC procedure** | **Issue**  **category** | **Subjects affected** | **Images affected** | **Action** |
| --- | --- | --- | --- | --- | --- | --- | --- |
| Input-image  QC | HC | — | dMRIqc | Input-image quality | 0 | 0 |  |
| Input-image  QC | MS | — | dMRIqc | Input-image quality | 0 | 0 |  |
| Input-image  QC | AD | — | MRIQC +  visual review | Input-image quality | 0 | 0 |  |
| Segmentation processing | AD | XSEC | FreeSurfer v7 | Processing failure | 1 | 1 | Segmentation failed |
| Segmentation processing | AD | LONG | FreeSurfer v7 | Processing failure | 1 | 1 | Segmentation failed |
| Segmentation processing | AD | XSEC | SAMSEG | Processing failure | 5 | 17 | Segmentation failed |
| Segmentation processing | AD | LONG | SAMSEG | Processing failure | 2 | 5 | Segmentation failed |
| Segmentation-output QC | AD | XSEC | SIENAX | Brain-extraction error causing inaccurate volume estimates | 5 | 11 | Reprocessed using the HD-BET-based procedure; no outputs excluded. |
| Segmentation-output QC | HC, MS, AD | XSEC/LONG | All other pipeline–mode combinations | Gross segmentation error requiring exclusion | 0 | 0 | No additional completed segmentation outputs were excluded |

**Table S1. Summary of input-image quality control, pipeline processing failures, and segmentation-output quality control across cohorts.** Counts are reported by cohort, pipeline, and processing mode. The five AD participants affected by FreeSurfer v7 and/or SAMSEG processing failures were excluded from the longitudinal analyses.

| **Pipeline** | **High-agreement contexts** | **Lower-agreement contexts** | **Interpretation** |
| --- | --- | --- | --- |
| FreeSurfer v6/v7 | GM in HC/MS/AD; strong agreement with FastSurfer | Some subcortical comparisons with SAMSEG/CAT12 | Versions generally concordant, but outputs not directly interchangeable with all methods |
| FastSurfer | Strong agreement with FreeSurfer | MS GM lower agreement | Expected FreeSurfer-like behavior; evaluated cross-sectionally only |
| SIENA | WM generally concordant; SIENA-XL useful for longitudinal GM/WM | GM agreement low for SIENAX in some cohorts | Interpret SIENAX/SIENA-XL as FSL-family estimates, not directly interchangeable with segmentation pipelines |
| SAMSEG | High repeatability | AD WM and subcortical divergence | Stable within pipeline but not always concordant with other methods |
| CAT12 | Some AD GM agreement | GM/subcortical systematic offsets | Atlas/tissue-definition differences likely contribute |
| SIENAX | Useful cross-sectional FSL estimate | Low GM agreement in HC/AD | Strong caution for GM comparisons |

**Table S2. Summary of observed inter-pipeline agreement patterns.** The table summarizes pairwise inter-pipeline agreement patterns based on intraclass correlation coefficients for normalized volume estimates across cohorts and tissues. Agreement categories are intended as descriptive summaries of concordance between pipeline outputs. Lower agreement may reflect differences in tissue definitions, atlas boundaries, preprocessing choices, or longitudinal modeling rather than incorrect segmentation. Context-specific discrepancies are highlighted to guide interpretation and to emphasize that outputs from different pipelines should not be treated as interchangeable.

| **Tissue** | **Mode** | **Pipeline** | **Standard Deviation**  **of change** | **Mean**  **of change** | **Standard Error Measurement** | **Bland-Altman Repeatability Coefficient** |
| --- | --- | --- | --- | --- | --- | --- |
| Grey matter | XSEC | CAT12 | 0.942 | 0.766 | 0.666 | 0.944 |
| Grey matter | XSEC | FreeSurfer v6 | 1.286 | 1.047 | 0.910 | 1.216 |
| Grey matter | XSEC | FreeSurfer v7 | 1.225 | 1.052 | 0.866 | 1.173 |
| Grey matter | XSEC | FastSurfer | 1.014 | 0.769 | 0.717 | 0.952 |
| Grey matter | XSEC | SAMSEG | 0.804 | 0.592 | 0.568 | 0.752 |
| Grey matter | XSEC | SIENA | 1.443 | 1.181 | 1.020 | 1.189 |
| Grey matter | LONG | CAT12 | 0.815 | 0.609 | 0.576 | 0.793 |
| Grey matter | LONG | FreeSurfer v6 | 1.225 | 0.959 | 0.866 | 1.166 |
| Grey matter | LONG | FreeSurfer v7 | 1.082 | 0.928 | 0.765 | 1.065 |
| Grey matter | LONG | SAMSEG | 0.898 | 0.749 | 0.635 | 0.907 |
| Grey matter | LONG | SIENA | 1.138 | 0.889 | 0.805 | 1.057 |
| White matter | XSEC | CAT12 | 0.572 | 0.452 | 0.404 | 0.373 |
| White matter | XSEC | FreeSurfer v6 | 0.783 | 0.549 | 0.554 | 0.456 |
| White matter | XSEC | FreeSurfer v7 | 0.848 | 0.625 | 0.599 | 0.534 |
| White matter | XSEC | FastSurfer | 0.527 | 0.403 | 0.372 | 0.356 |
| White matter | XSEC | SAMSEG | 0.539 | 0.430 | 0.381 | 0.357 |
| White matter | XSEC | SIENA | 0.850 | 0.699 | 0.601 | 0.623 |
| White matter | LONG | CAT12 | 0.487 | 0.404 | 0.344 | 0.320 |
| White matter | LONG | FreeSurfer v6 | 0.648 | 0.499 | 0.458 | 0.394 |
| White matter | LONG | FreeSurfer v7 | 0.549 | 0.467 | 0.388 | 0.339 |
| White matter | LONG | SAMSEG | 0.483 | 0.421 | 0.342 | 0.416 |
| White matter | LONG | SIENA | 1.285 | 1.006 | 0.909 | 0.774 |
| Hippocampus | XSEC | CAT12 | 0.895 | 0.715 | 0.633 | 0.009 |
| Hippocampus | XSEC | FreeSurfer v6 | 1.936 | 1.468 | 1.369 | 0.019 |
| Hippocampus | XSEC | FreeSurfer v7 | 2.235 | 1.968 | 1.581 | 0.026 |
| Hippocampus | XSEC | FastSurfer | 0.918 | 0.830 | 0.649 | 0.011 |
| Hippocampus | XSEC | SAMSEG | 1.027 | 0.844 | 0.726 | 0.012 |
| Hippocampus | LONG | CAT12 | 0.728 | 0.612 | 0.515 | 0.007 |
| Hippocampus | LONG | FreeSurfer v6 | 0.944 | 0.772 | 0.668 | 0.011 |
| Hippocampus | LONG | FreeSurfer v7 | 0.686 | 0.584 | 0.485 | 0.010 |
| Hippocampus | LONG | SAMSEG | 0.666 | 0.537 | 0.471 | 0.009 |
| Thalamus | XSEC | CAT12 | 1.223 | 0.997 | 0.865 | 0.025 |
| Thalamus | XSEC | FreeSurfer v6 | 3.239 | 2.400 | 2.291 | 0.057 |
| Thalamus | XSEC | FreeSurfer v7 | 2.726 | 2.140 | 1.928 | 0.053 |
| Thalamus | XSEC | FastSurfer | 1.138 | 0.891 | 0.805 | 0.024 |
| Thalamus | XSEC | SAMSEG | 0.913 | 0.757 | 0.645 | 0.015 |
| Thalamus | LONG | CAT12 | 1.823 | 1.509 | 1.289 | 0.038 |
| Thalamus | LONG | FreeSurfer v6 | 1.025 | 0.822 | 0.725 | 0.020 |
| Thalamus | LONG | FreeSurfer v7 | 1.050 | 0.841 | 0.742 | 0.020 |
| Thalamus | LONG | SAMSEG | 0.580 | 0.475 | 0.410 | 0.010 |

**Table S3**. **Repeatability metrics estimated in the Healthy Control cohort.** All repeatability metrics were estimated in the healthy control cohort only. The standard deviation of change and mean absolute change were calculated using follow-up change-from-baseline estimates. The standard error of measurement was calculated as SD change divided by √2. The Bland–Altman repeatability coefficient was estimated using all available healthy-control timepoints. For each tissue, processing mode, and pipeline, a random-intercept model was fitted to normalized volume estimates with subject as a random effect. The residual standard deviation was interpreted as the within-subject standard deviation, and the repeatability coefficient was calculated as 1.96 × √2 × within-subject SD.

|  |  |  |  |  | **Relative percentage change** | | | **Signed change in %ICV points** | | |
| --- | --- | --- | --- | --- | --- | --- | --- | --- | --- | --- |
| **Cohort** | | **Tissue** | **Mode** | **Term** | **F** | **p** | **FDR p** | **F** | **p** | **FDR p** |
| HC | | Grey matter | XSEC | Pipeline | 1.58 | 0.16 | 0.22 | 1.56 | 0.17 | 0.22 |
|  | |  |  | Timepoint | 2.61 | 0.05 | 0.09 | 2.72 | 0.04 | 0.08 |
|  | |  |  | Pipeline:Timepoint | 0.28 | 1.00 | 1.00 | 0.26 | 1.00 | 1.00 |
| HC | | Grey matter | LONG | Pipeline | 2.33 | 0.06 | 0.10 | 2.23 | 0.07 | 0.11 |
|  | |  |  | Timepoint | 0.41 | 0.75 | 0.75 | 0.41 | 0.75 | 0.75 |
|  | |  |  | Pipeline:Timepoint | 0.21 | 1.00 | 1.00 | 0.21 | 1.00 | 1.00 |
| **HC** | | **White matter** | **XSEC** | **Pipeline** | **2.58** | **0.03** | **0.06** | **2.56** | **0.03** | **0.05** |
|  | |  |  | Timepoint | 2.05 | 0.11 | 0.17 | 1.81 | 0.14 | 0.22 |
|  | |  |  | Pipeline:Timepoint | 0.25 | 1.00 | 1.00 | 0.25 | 1.00 | 1.00 |
| **HC** | | **White matter** | **LONG** | **Pipeline** | **4.41** | **0.002** | **0.005** | **4.53** | **0.001** | **0.003** |
|  | |  |  | Timepoint | 1.24 | 0.30 | 0.40 | 1.09 | 0.35 | 0.45 |
|  | |  |  | Pipeline:Timepoint | 0.76 | 0.69 | 1.00 | 0.77 | 0.68 | 1.00 |
| **HC** | | **Hippocampus** | **XSEC** | **Pipeline** | **4.37** | **0.002** | **0.005** | **4.81** | **<0.001** | **0.002** |
|  | |  |  | Timepoint | 0.73 | 0.53 | 0.58 | 0.72 | 0.54 | 0.59 |
|  | |  |  | Pipeline:Timepoint | 0.42 | 0.95 | 1.00 | 0.39 | 0.97 | 1.00 |
| HC | | Hippocampus | LONG | Pipeline | 1.93 | 0.13 | 0.20 | 1.81 | 0.15 | 0.20 |
|  | |  |  | Timepoint | 1.89 | 0.13 | 0.20 | 1.87 | 0.13 | 0.22 |
|  | |  |  | Pipeline:Timepoint | 0.12 | 1.00 | 1.00 | 0.10 | 1.00 | 1.00 |
| HC | | Thalamus | XSEC | Pipeline | 1.05 | 0.38 | 0.46 | 1.41 | 0.23 | 0.28 |
|  | |  |  | Timepoint | 1.04 | 0.38 | 0.45 | 0.98 | 0.40 | 0.46 |
|  | |  |  | Pipeline:Timepoint | 0.27 | 0.99 | 1.00 | 0.29 | 0.99 | 1.00 |
| HC | | Thalamus | LONG | Pipeline | 0.45 | 0.72 | 0.72 | 0.57 | 0.63 | 0.66 |
|  | |  |  | Timepoint | 2.88 | 0.04 | 0.07 | 2.80 | 0.04 | 0.08 |
|  | |  |  | Pipeline:Timepoint | 0.84 | 0.58 | 1.00 | 0.92 | 0.51 | 1.00 |
| MS | | Grey matter | XSEC | Pipeline | 2.20 | 0.05 | 0.10 | 2.29 | 0.05 | 0.08 |
|  | |  |  | Timepoint | 4.89 | 0.002 | 0.006 | 5.15 | 0.002 | 0.004 |
|  | |  |  | Pipeline:Timepoint | 0.24 | 1.00 | 1.00 | 0.22 | 1.00 | 1.00 |
| MS | | Grey matter | LONG | Pipeline | 1.78 | 0.13 | 0.20 | 1.80 | 0.13 | 0.19 |
|  | |  |  | Timepoint | 3.69 | 0.01 | 0.03 | 3.77 | 0.01 | 0.02 |
|  | |  |  | Pipeline:Timepoint | 0.24 | 1.00 | 1.00 | 0.24 | 1.00 | 1.00 |
| MS | | White matter | XSEC | Pipeline | 0.88 | 0.49 | 0.54 | 0.89 | 0.48 | 0.53 |
|  | |  |  | Timepoint | 1.39 | 0.24 | 0.35 | 1.72 | 0.16 | 0.23 |
|  | |  |  | Pipeline:Timepoint | 0.32 | 0.99 | 1.00 | 0.31 | 0.99 | 1.00 |
| MS | | White matter | LONG | Pipeline | 1.64 | 0.16 | 0.22 | 1.76 | 0.14 | 0.19 |
|  | |  |  | Timepoint | 0.77 | 0.51 | 0.58 | 0.99 | 0.40 | 0.46 |
|  | |  |  | Pipeline:Timepoint | 0.54 | 0.89 | 1.00 | 0.49 | 0.92 | 1.00 |
| **MS** | | **Hippocampus** | **XSEC** | **Pipeline** | **6.10** | **<0.001** | **<0.001** | **5.41** | **<0.001** | **<0.001** |
|  |  |  |  | Timepoint | 4.01 | 0.008 | 0.02 | 4.09 | 0.007 | 0.02 |
|  |  |  |  | Pipeline:Timepoint | 0.69 | 0.76 | 1.00 | 0.72 | 0.73 | 1.00 |
|  | **MS** | **Hippocampus** | **LONG** | **Pipeline** | **2.87** | **0.04** | **0.07** | **3.10** | **0.03** | **0.05** |
|  |  |  |  | Timepoint | 2.08 | 0.10 | 0.17 | 2.25 | 0.08 | 0.14 |
|  |  |  |  | Pipeline:Timepoint | 0.30 | 0.97 | 1.00 | 0.34 | 0.96 | 1.00 |
|  | MS | Thalamus | XSEC | Pipeline | 0.96 | 0.43 | 0.49 | 0.98 | 0.42 | 0.48 |
|  |  |  |  | Timepoint | 1.06 | 0.37 | 0.45 | 1.08 | 0.36 | 0.45 |
|  |  |  |  | Pipeline:Timepoint | 0.77 | 0.68 | 1.00 | 0.77 | 0.68 | 1.00 |
|  | MS | Thalamus | LONG | Pipeline | 1.49 | 0.22 | 0.27 | 1.90 | 0.13 | 0.19 |
|  |  |  |  | Timepoint | 0.47 | 0.70 | 0.73 | 0.53 | 0.66 | 0.69 |
|  |  |  |  | Pipeline:Timepoint | 0.28 | 0.98 | 1.00 | 0.25 | 0.99 | 1.00 |
|  | AD | Grey matter | XSEC | Pipeline | 0.74 | 0.59 | 0.62 | 0.32 | 0.90 | 0.90 |
|  |  |  |  | Timepoint | 70.16 | <0.001 | <0.001 | 96.78 | <0.001 | <0.001 |
|  |  |  |  | Pipeline:Timepoint | 0.37 | 0.96 | 1.00 | 0.16 | 1.00 | 1.00 |
|  | **AD** | **Grey matter** | **LONG** | **Pipeline** | **8.38** | **<0.001** | **<0.001** | **10.42** | **<0.001** | **<0.001** |
|  |  |  |  | Timepoint | 124.90 | <0.001 | <0.001 | 125.90 | <0.001 | <0.001 |
|  |  |  |  | Pipeline:Timepoint | 1.00 | 0.43 | 1.00 | 1.11 | 0.35 | 1.00 |
|  | **AD** | **White matter** | **XSEC** | **Pipeline** | **20.47** | **<0.001** | **<0.001** | **19.53** | **<0.001** | **<0.001** |
|  |  |  |  | Timepoint | 24.03 | <0.001 | <0.001 | 23.66 | <0.001 | <0.001 |
|  |  |  |  | Pipeline:Timepoint | 1.61 | 0.10 | 1.00 | 1.50 | 0.13 | 1.00 |
|  | **AD** | **White matter** | **LONG** | **Pipeline** | **39.92** | **<0.001** | **<0.001** | **45.68** | **<0.001** | **<0.001** |
|  |  |  |  | Timepoint | 16.01 | <0.001 | <0.001 | 16.55 | <0.001 | <0.001 |
|  |  |  |  | Pipeline:Timepoint | 0.24 | 0.98 | 1.00 | 0.25 | 0.98 | 1.00 |
|  | **AD** | **Hippocampus** | **XSEC** | **Pipeline** | **11.86** | **<0.001** | **<0.001** | **5.53** | **<0.001** | **<0.001** |
|  |  |  |  | Timepoint | 232.53 | <0.001 | <0.001 | 234.39 | <0.001 | <0.001 |
|  |  |  |  | Pipeline:Timepoint | 1.28 | 0.25 | 1.00 | 0.67 | 0.71 | 1.00 |
|  | **AD** | **Hippocampus** | **LONG** | **Pipeline** | **24.21** | **<0.001** | **<0.001** | **9.49** | **<0.001** | **<0.001** |
|  |  |  |  | Timepoint | 347.39 | <0.001 | <0.001 | 337.00 | <0.001 | <0.001 |
|  |  |  |  | Pipeline:Timepoint | 0.75 | 0.61 | 1.00 | 0.42 | 0.87 | 1.00 |
|  | **AD** | **Thalamus** | **XSEC** | **Pipeline** | **8.56** | **<0.001** | **<0.001** | **10.13** | **<0.001** | **<0.001** |
|  |  |  |  | Timepoint | 32.24 | <0.001 | <0.001 | 32.03 | <0.001 | <0.001 |
|  |  |  |  | Pipeline:Timepoint | 1.23 | 0.28 | 1.00 | 1.33 | 0.23 | 1.00 |
|  | **AD** | **Thalamus** | **LONG** | **Pipeline** | **10.05** | **<0.001** | **<0.001** | **7.16** | **<0.001** | **<0.001** |
|  |  |  |  | Timepoint | 47.46 | <0.001 | <0.001 | 41.89 | <0.001 | <0.001 |
|  |  |  |  | Pipeline:Timepoint | 0.34 | 0.91 | 1.00 | 0.71 | 0.64 | 1.00 |

**Table S4. Longitudinal mixed-effects models using relative percentage change and signed change in %ICV points.** Models were fitted separately by cohort, tissue, and processing mode with Pipeline, Timepoint, and Pipeline × Timepoint as fixed effects and participant as a random intercept. Relative change was calculated as 100×(V_follow-up_−V_baseline_)/V_baseline_. To assess whether pipeline-dependent baseline offsets influenced results through the denominator, models were repeated using signed change in normalized volume, V_follow-up_−V_baseline_, expressed in %ICV points. P-values were FDR-adjusted separately by model term across cohort, tissue, and processing-mode models.

| **Cohort** | **Tissue** | **Pipeline 1** | **Pipeline 2** | **r** | **FDR-adjusted**  **p-value** |
| --- | --- | --- | --- | --- | --- |
| AD | White matter | CAT12 LONG | SAMSEG LONG | 0.37 | 0.01 |
| AD | White matter | CAT12 XSEC | SAMSEG LONG | 0.40 | 0.01 |
| AD | White matter | SAMSEG LONG | SIENA XSEC | 0.28 | 0.06 |

**Table S5. Pairwise Pearson correlations for volume estimates at baseline.** Pearson’s r values and their associated pFDR-adjusted p-values are listed here for pipeline pairings where values were not equal or smaller than 0.001.

| **Mode** | **Pipeline** | **Week** | **Mean change from week 4**  **%ICV (95% CI)** |
| --- | --- | --- | --- |
| XSEC | CAT12 | 4 | 0.00 (0.00, 0.00) |
| XSEC | CAT12 | 8 | -0.28 (-0.97, 0.41) |
| XSEC | CAT12 | 12 | 0.08 (-0.60, 0.76) |
| XSEC | CAT12 | 16 | -0.26 (-0.92, 0.39) |
| XSEC | FreeSurfer v6 | 4 | 0.00 (0.00, 0.00) |
| XSEC | FreeSurfer v6 | 8 | -0.16 (-0.96, 0.64) |
| XSEC | FreeSurfer v6 | 12 | 0.01 (-1.10, 1.12) |
| XSEC | FreeSurfer v6 | 16 | -0.07 (-0.81, 0.68) |
| XSEC | FreeSurfer v7 | 4 | 0.00 (0.00, 0.00) |
| XSEC | FreeSurfer v7 | 8 | -0.11 (-0.82, 0.59) |
| XSEC | FreeSurfer v7 | 12 | 0.05 (-1.03, 1.13) |
| XSEC | FreeSurfer v7 | 16 | 0.03 (-0.74, 0.81) |
| XSEC | FastSurfer | 4 | 0.00 (0.00, 0.00) |
| XSEC | FastSurfer | 8 | -0.18 (-0.90, 0.54) |
| XSEC | FastSurfer | 12 | 0.39 (-0.51, 1.28) |
| XSEC | FastSurfer | 16 | 0.07 (-0.70, 0.83) |
| XSEC | SAMSEG | 4 | 0.00 (0.00, 0.00) |
| XSEC | SAMSEG | 8 | -0.03 (-0.61, 0.55) |
| XSEC | SAMSEG | 12 | 0.18 (-0.51, 0.86) |
| XSEC | SAMSEG | 16 | 0.06 (-0.44, 0.57) |
| XSEC | SIENA | 4 | 0.00 (0.00, 0.00) |
| XSEC | SIENA | 8 | -0.53 (-1.32, 0.26) |
| XSEC | SIENA | 12 | 0.16 (-0.77, 1.08) |
| XSEC | SIENA | 16 | -0.36 (-1.21, 0.49) |
| LONG | CAT12 | 4 | 0.00 (0.00, 0.00) |
| LONG | CAT12 | 8 | -0.25 (-0.87, 0.37) |
| LONG | CAT12 | 12 | -0.00 (-0.70, 0.69) |
| LONG | CAT12 | 16 | -0.20 (-0.77, 0.36) |
| LONG | FreeSurfer v6 | 4 | 0.00 (0.00, 0.00) |
| LONG | FreeSurfer v6 | 8 | -0.08 (-0.94, 0.77) |
| LONG | FreeSurfer v6 | 12 | -0.07 (-1.10, 0.97) |
| LONG | FreeSurfer v6 | 16 | -0.08 (-0.87, 0.72) |
| LONG | FreeSurfer v7 | 4 | 0.00 (0.00, 0.00) |
| LONG | FreeSurfer v7 | 8 | -0.15 (-0.91, 0.61) |
| LONG | FreeSurfer v7 | 12 | 0.11 (-0.87, 1.08) |
| LONG | FreeSurfer v7 | 16 | 0.11 (-0.58, 0.81) |
| LONG | SAMSEG | 4 | 0.00 (0.00, 0.00) |
| LONG | SAMSEG | 8 | -0.01 (-0.79, 0.77) |
| LONG | SAMSEG | 12 | 0.30 (-0.52, 1.13) |
| LONG | SAMSEG | 16 | -0.01 (-0.69, 0.68) |
| LONG | SIENA | 4 | 0.00 (0.00, 0.00) |
| LONG | SIENA | 8 | -0.01 (-0.71, 0.70) |
| LONG | SIENA | 12 | -0.13 (-0.93, 0.67) |
| LONG | SIENA | 16 | -0.09 (-0.91, 0.74) |

**Table S6. Grey matter change in HC cohort after re-baselining at week 4.**

| **Mode** | **Pipeline** | **Week** | **Mean bias,  %ICV** | **95% LoA,  %ICV** |
| --- | --- | --- | --- | --- |
| LONG | CAT12 | 4 | -0.03 | -0.95 to 0.90 |
| LONG | CAT12 | 8 | -0.14 | -0.69 to 0.41 |
| LONG | CAT12 | 12 | -0.03 | -0.81 to 0.75 |
| LONG | CAT12 | 16 | -0.12 | -0.63 to 0.39 |
| LONG | FreeSurfer v6 | 4 | -0.19 | -1.46 to 1.07 |
| LONG | FreeSurfer v6 | 8 | -0.23 | -1.16 to 0.70 |
| LONG | FreeSurfer v6 | 12 | -0.22 | -1.51 to 1.07 |
| LONG | FreeSurfer v6 | 16 | -0.23 | -0.88 to 0.42 |
| LONG | FreeSurfer v7 | 4 | -0.26 | -1.19 to 0.68 |
| LONG | FreeSurfer v7 | 8 | -0.32 | -1.17 to 0.53 |
| LONG | FreeSurfer v7 | 12 | -0.21 | -1.40 to 0.98 |
| LONG | FreeSurfer v7 | 16 | -0.21 | -0.85 to 0.43 |
| LONG | SAMSEG | 4 | -0.26 | -1.41 to 0.88 |
| LONG | SAMSEG | 8 | -0.27 | -0.73 to 0.19 |
| LONG | SAMSEG | 12 | -0.14 | -0.77 to 0.50 |
| LONG | SAMSEG | 16 | -0.27 | -0.89 to 0.36 |
| LONG | SIENA | 4 | -0.22 | -1.44 to 1.00 |
| LONG | SIENA | 8 | -0.23 | -0.83 to 0.38 |
| LONG | SIENA | 12 | -0.28 | -1.38 to 0.82 |
| LONG | SIENA | 16 | -0.26 | -1.23 to 0.70 |
| XSEC | CAT12 | 4 | -0.11 | -1.14 to 0.92 |
| XSEC | CAT12 | 8 | -0.24 | -1.05 to 0.57 |
| XSEC | CAT12 | 12 | -0.07 | -0.79 to 0.65 |
| XSEC | CAT12 | 16 | -0.23 | -1.05 to 0.59 |
| XSEC | FastSurfer | 4 | -0.19 | -1.42 to 1.05 |
| XSEC | FastSurfer | 8 | -0.26 | -0.96 to 0.44 |
| XSEC | FastSurfer | 12 | -0.03 | -0.74 to 0.69 |
| XSEC | FastSurfer | 16 | -0.16 | -0.60 to 0.28 |
| XSEC | FreeSurfer v6 | 4 | -0.23 | -1.56 to 1.11 |
| XSEC | FreeSurfer v6 | 8 | -0.29 | -1.17 to 0.58 |
| XSEC | FreeSurfer v6 | 12 | -0.21 | -1.57 to 1.14 |
| XSEC | FreeSurfer v6 | 16 | -0.26 | -0.98 to 0.47 |
| XSEC | FreeSurfer v7 | 4 | -0.18 | -1.40 to 1.03 |
| XSEC | FreeSurfer v7 | 8 | -0.23 | -1.02 to 0.55 |
| XSEC | FreeSurfer v7 | 12 | -0.16 | -1.42 to 1.09 |
| XSEC | FreeSurfer v7 | 16 | -0.17 | -1.00 to 0.65 |
| XSEC | SAMSEG | 4 | -0.10 | -0.96 to 0.77 |
| XSEC | SAMSEG | 8 | -0.11 | -0.64 to 0.42 |
| XSEC | SAMSEG | 12 | -0.02 | -0.76 to 0.72 |
| XSEC | SAMSEG | 16 | -0.07 | -0.62 to 0.47 |
| XSEC | SIENA | 4 | -0.15 | -1.37 to 1.07 |
| XSEC | SIENA | 8 | -0.37 | -1.41 to 0.67 |
| XSEC | SIENA | 12 | -0.09 | -1.26 to 1.09 |
| XSEC | SIENA | 16 | -0.30 | -1.44 to 0.84 |

**Table S7. Bland-Altman analysis of healthy-control grey matter differences between baseline and follow-up.** Bias was calculated as follow-up minus baseline with negative values indicating a lower follow-up grey matter estimate. Limits of agreement were calculated as the mean bias +/- 1.96 standard deviations of the individual differences. ICV, intracranial volume; LoA, limits of agreement.

| **Target** | **Tissue** | **Mappings,  n** | **Native absolute bias,  median (IQR)** | **Calibrated absolute bias,  median (IQR)** | **Native MAE,  median (IQR)** | **Calibrated MAE,  median (IQR)** | **Native CCC,  median (IQR)** | **Calibrated CCC,  median (IQR)** | **MAE improved,  %** |
| --- | --- | --- | --- | --- | --- | --- | --- | --- | --- |
| **MS** | **Overall** | **82** | **0.13 (0.06-**  **0.91)** | **0.08 (0.02-**  **0.26)** | **0.29 (0.08-**  **0.99)** | **0.26 (0.03-**  **0.55)** | **0.71 (0.44-**  **0.90)** | **0.90 (0.72-**  **0.96)** | **82.93** |
| MS | Grey matter | 25 | 1.22 (0.80-  2.26) | 0.26 (0.07-  0.44) | 1.30 (0.84-  2.26) | 0.63 (0.43-  0.69) | 0.56 (0.23-  0.75) | 0.76 (0.71-  0.90) | 92.00 |
| MS | White matter | 25 | 0.44 (0.09-  0.67) | 0.19 (0.08-  0.28) | 0.65 (0.26-  0.80) | 0.30 (0.26-  0.52) | 0.95 (0.86-  0.99) | 0.98 (0.93-  0.99) | 80.00 |
| MS | Hippocampus | 16 | 0.04 (0.02-  0.09) | 0.01 (0.00-  0.02) | 0.04 (0.02-  0.09) | 0.02 (0.01-  0.03) | 0.45 (0.18-  0.71) | 0.88 (0.67-  0.92) | 87.50 |
| MS | Thalamus | 16 | 0.06 (0.04-  0.09) | 0.01 (0.01-  0.08) | 0.06 (0.05-  0.09) | 0.03 (0.02-  0.10) | 0.66 (0.49-  0.80) | 0.81 (0.60-  0.90) | 68.75 |
| **AD** | **Overall** | **82** | **0.32 (0.07-**  **1.32)** | **0.30 (0.03-**  **1.39)** | **0.74 (0.07-**  **1.59)** | **0.86 (0.04-**  **1.80)** | **0.71 (0.47-**  **0.89)** | **0.78 (0.61-**  **0.89)** | **60.98** |
| AD | Grey matter | 25 | 1.19 (0.61-  2.25) | 1.15 (0.69-  1.58) | 1.39 (0.75-  2.25) | 1.35 (0.93-  1.93) | 0.79 (0.67-  0.92) | 0.81 (0.69-  0.89) | 48.00 |
| AD | White matter | 25 | 1.20 (0.38-  1.70) | 1.17 (0.27-  2.35) | 1.50 (1.07-  2.06) | 1.80 (1.04-  2.43) | 0.78 (0.66-  0.83) | 0.71 (0.52-  0.85) | 48.00 |
| AD | Hippocampus | 16 | 0.05 (0.02-  0.07) | 0.01 (0.01-  0.03) | 0.05 (0.03-  0.07) | 0.02 (0.02-  0.04) | 0.69 (0.51-  0.85) | 0.85 (0.77-  0.93) | 75.00 |
| AD | Thalamus | 16 | 0.08 (0.04-  0.11) | 0.02 (0.01-  0.04) | 0.08 (0.04-  0.11) | 0.03 (0.03-  0.06) | 0.43 (0.32-  0.75) | 0.67 (0.62-  0.79) | 87.50 |

**Table S8. Summary of HC-derived calibration performance after transfer to MS and AD.** The table reports medians, with interquartile ranges shown as the 25th-75th percentiles, across all available pipeline-pair and processing-mode mappings within each tissue. Pairwise Deming equations were estimated using HC baseline data and applied without refitting to baseline measurements in the target cohort. Absolute bias and MAE are expressed in percentage points of ICV. 'MAE improved' represents the percentage of mappings for which calibrated MAE was lower than native MAE. CCC, Lin's concordance correlation coefficient; ICV, intracranial volume; IQR, interquartile range; MAE, mean absolute error.

# Figures

**Figure S1. Pairwise Pearson correlations for hippocampus and thalamus volume estimates at baseline.** Heatmaps show pairwise Pearson correlation coefficients between baseline normalized volume estimates from cross-sectional and longitudinal implementations of each pipeline, stratified by cohort and tissue. Values inside tiles indicate Pearson’s r. The FDR-adjusted p-values were < 0.001 for all the pairwise comparisons. Higher correlations indicate stronger linear association between pipeline-derived estimates but do not imply agreement in absolute values or segmentation accuracy.

**Figure S2. Intra-pipeline agreement across all healthy control timepoints.** Intraclass correlation coefficients (ICCs) were calculated separately for each pipeline, processing mode, and tissue using normalized volume estimates from all available healthy control timepoints. Points show ICC estimates, and vertical bars show 95% confidence intervals. The dashed horizontal line indicates the threshold for excellent agreement (ICC ≥ 0.90). Blue points indicate repeated cross-sectional processing, and green points indicate longitudinal processing. Because the healthy control cohort was not expected to show substantial biological or pathology-related change over the short follow-up interval, this analysis was used as an estimate of within-pipeline measurement stability over time. Higher ICC values indicate greater within-pipeline consistency but should not be interpreted as evidence of segmentation accuracy.

**Figure S3. Inter-pipeline agreement of normalized volume estimates at baseline in subcortical regions.** Heatmaps show pairwise ICCs for absolute agreement between pipeline outputs at baseline, stratified by tissue and cohort. Cross-sectional and longitudinal implementations are displayed as separate pipeline-mode outputs where available. Higher ICC values are represented in blue and lower in red.

**Figure S4. Longitudinal change-from-baseline estimates by segmentation pipeline in subcortical regions.** Mean percentage change from baseline is shown over time for each pipeline, stratified by tissue, processing mode, and cohort. Error bars represent the standard error of the mean across participants. The horizontal reference line indicates no change from baseline.

**Figure S5. Participant-level grey-matter trajectories in healthy controls.** Percentage change in grey matter volume from the original baseline is shown across the five study visits (weeks 0, 4, 8, 12, and 16) for (A) cross-sectional and (B) longitudinal processing modes. Thin lines represent individual participants, while thick lines and points indicate the pipeline-specific mean at each visit; error bars represent 95% confidence intervals. The horizontal dashed line indicates no change from baseline. FastSurfer is shown only for the cross-sectional mode because no longitudinal FastSurfer processing was performed.
